# Marine Biopolymers as Bioactive Functional Ingredients of Electrospun Nanofibrous Scaffolds for Biomedical Applications

**DOI:** 10.3390/md20050314

**Published:** 2022-05-05

**Authors:** Konstantina Iliou, Stefanos Kikionis, Efstathia Ioannou, Vassilios Roussis

**Affiliations:** Section of Pharmacognosy and Chemistry of Natural Products, Department of Pharmacy, National and Kapodistrian University of Athens, Panepistimiopolis Zografou, 15771 Athens, Greece; ilioukonstantina@outlook.com (K.I.); skikionis@pharm.uoa.gr (S.K.); eioannou@pharm.uoa.gr (E.I.)

**Keywords:** marine biopolymers, electrospun nanofibers, tissue regeneration, drug delivery, wound healing

## Abstract

Marine biopolymers, abundantly present in seaweeds and marine animals, feature diverse structures and functionalities, and possess a wide range of beneficial biological activities. Characterized by high biocompatibility and biodegradability, as well as unique physicochemical properties, marine biopolymers are attracting a constantly increasing interest for the development of advanced systems for applications in the biomedical field. The development of electrospinning offers an innovative technological platform for the production of nonwoven nanofibrous scaffolds with increased surface area, high encapsulation efficacy, intrinsic interconnectivity, and structural analogy to the natural extracellular matrix. Marine biopolymer-based electrospun nanofibrous scaffolds with multifunctional characteristics and tunable mechanical properties now attract significant attention for biomedical applications, such as tissue engineering, drug delivery, and wound healing. The present review, covering the literature up to the end of 2021, highlights the advancements in the development of marine biopolymer-based electrospun nanofibers for their utilization as cell proliferation scaffolds, bioadhesives, release modifiers, and wound dressings.

## 1. Introduction

The marine ecosystem, due to its high biodiversity, represents a plentiful source of bioactive compounds with diverse structures and biological properties [1,2]. Among them, biopolymers, including polysaccharides and proteins, are abundantly found in all marine organisms, serving biological and structural functions. They are often isolated from a variety of marine organisms, including algae, crustaceans, fish, shellfish, mollusks, and microorganisms, and have been extensively investigated for their potential applications in various sectors [1,2,3]. In recent years, there has been an increasing interest in the biomedical sector for marine biopolymers due to their diversified chemical structures and functionalities which, in turn, attenuate their biological and physicochemical properties [4,5,6,7]. Characterized by low immunogenicity, remarkable bioavailability, chelating ability, and stability, marine-derived polymers often represent materials of choice for biomedical applications. The broad spectrum of their bioactivities—such as anticoagulant, antioxidant, antimicrobial, anticancer, and cell proliferative effects—and their excellent biocompatibility and biodegradability make them ideal candidates as safe biomaterials for the development of value-added biomedical systems for tissue regeneration, drug delivery, and wound healing applications [2,3,8].

**Figure 1:**
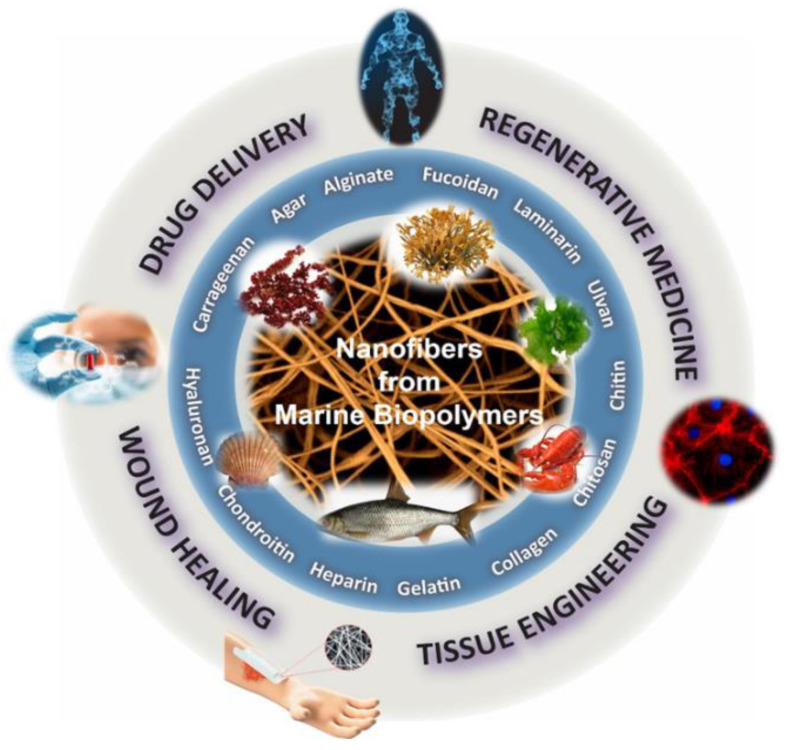
Biomedical applications of marine biopolymer-based nanofibers.

Over the last years, polymeric nanofibers have attracted significant interest in the biomedical sector due to their high surface-to-volume area, high porosity, and tunable mechanical properties [9,10,11]. Exhibiting distinctive characteristics, such as high encapsulation efficacy, intrinsic interconnectivity, and structural analogy to the natural extracellular matrix (ECM), they can be used as scaffolds to promote cell growth and serve as carriers of bioactive agents. Electrospinning is the most efficient and frequently used technique for the preparation of ultrafine polymeric fibers [12,13,14]. Electrospinning is a highly versatile, simple, and cost-effective method for the production of polymeric fibers with diameters ranging from submicrons down to the nanometer scale. Its basic laboratory-scale setup includes a high-voltage power supply, a grounded collector, a syringe pump, and a spinneret coupled to a syringe filled with a polymer solution or melt. Under the application of high voltage at the tip of the spinneret, the charged polymer is ejected from the spinneret, stretched under electrostatic repulsion, solidified after evaporation of the solvent, and deposited on a grounded collector in the form of a fibrous mat. Depending on the selection of materials (e.g., polymers, solvents, active ingredients), solution properties (e.g., concentration, conductivity, surface tension), and electrospinning parameters (e.g., applied voltage, polymer flow rate, tip-to-collector distance), fibers with diverse morphologies and tailor-made properties can be produced [15,16,17,18,19]. Numerous polymeric materials, excipients, and active pharmaceutical ingredients (APIs) can be formulated into nanofibrous nonwovens for different biomedical applications. Among them, marine biopolymers formulated in nanofibrous structures have been extensively investigated as cell scaffolds, bioadhesives, release modifiers, and wound dressings for regenerative medicine, drug delivery, and wound healing applications (Figure 1).

**Figure 2:**
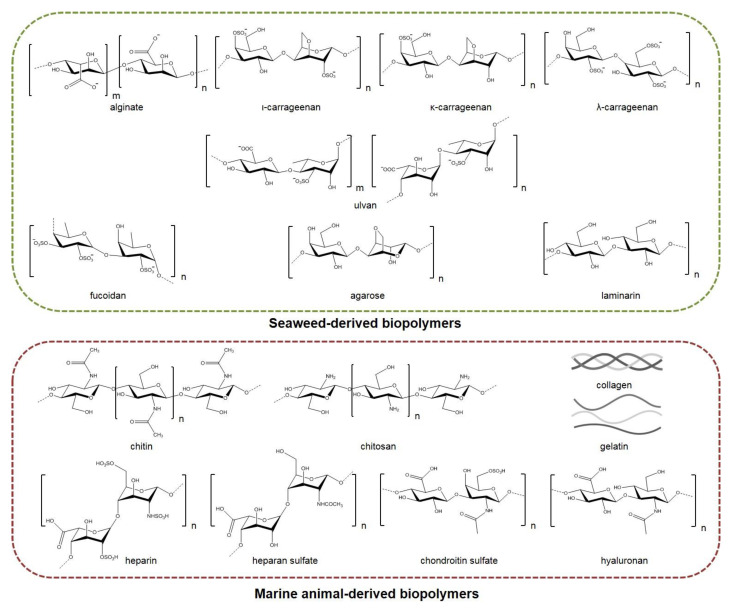
Seaweed- and marine animal-derived biopolymers used in the fabrication of electrospun nanofibrous scaffolds for biomedical applications.

The incorporation of marine biopolymers (Figure 2)—often lacking polymer chain entanglement—in electrospun nonwovens is a challenging process that usually requires them to be co-electrospun with other natural or synthetic polymers of greater electrospinnability. Polymeric blends, including dispersions, emulsions, and binary spinning solutions, have been electrospun using single or multiple needles, as well as coaxial and needleless electrospinning techniques, among others, for the preparation of marine-based nanofibrous scaffolds. Polyethylene oxide (PEO), polyvinyl alcohol (PVA), polycaprolactone (PCL), polylactic acid (PLA), and cellulose acetate have been widely used as polymer carriers to facilitate the electrospinnability of marine biopolymers, resulting in the development of hybrid nanofibrous scaffolds with multifunctional characteristics and enhanced mechanical properties. Such hybrid fibrous systems that combine the beneficial properties of the blended ingredients possess high potential for the development of novel tissue regeneration, wound healing, and controlled drug delivery systems [18,19,20].

The low electrospinnability of marine biopolymers is also associated with their polyelectrolyte character and high gelation. The spinning solutions of marine biopolymers are usually characterized by high electric conductivity, high viscosity, and high surface tension, which limit their spinning processability. The addition of surfactants—such as Triton-X-100, Pluronic F127, and lecithin—in the spinning solutions is a common strategy to reduce surface tension, increase biopolymer content, and facilitate the production of smooth nanofibers devoid of beads [21,22,23]. Another limitation concerning the application of marine biopolymeric fibers is their usually poor mechanical properties and fast degradation in aqueous media. Due to the hydrophilic nature of most marine biopolymers, various crosslinking treatments are often applied to provide the desired mechanical stability for applications where longevity in aqueous media is necessary. Different physical and chemical crosslinking methodologies have been reported in the literature, with polyelectrolyte complexation and exposure to glutaraldehyde vapors being the most widely employed crosslinking strategies to stabilize and reinforce marine-based nanofibrous scaffolds [21,22,23,24].

Herein, marine biopolymers that have been used in the fabrication of electrospun nanofibers are presented, along with their general characteristics and potential for biomedical applications. This review, covering the literature until December 2021, aims to highlight the advancements in the development of electrospun marine biopolymer-based nanofibrous matrices for their application in the biomedical field as drug delivery systems, wound healing materials, and tissue engineering scaffolds.

## 2. Seaweed-Derived Biopolymers

### 2.1. Alginates

Alginates, including alginic acid and its respective salts, are a group of linear anionic polysaccharides isolated from the cell walls of brown algae, such as species of the genera *Ascophyllum*, *Laminaria*, *Lessonia*, and *Macrocystis*, but can also be isolated from bacteria of the genera *Azotobacter* and *Pseudomonas* [22,25]. Their chemical structures consist of 1–4 linked β-d-mannuronic (M) and α-l-guluronic (G) acid units forming either homopolymeric or heteropolymeric blocks [26,27,28]. The proportion and sequence of M/G units vary between the seaweed species, as well as by the location and season of collection which, in turn, affect the physicochemical properties of alginates [28,29,30]. Alginates are biostable, non-toxic hydrophilic polymers possessing stabilizing, thickening, and emulsifying properties. In particular, gelation of alginates occurs in the presence of divalent cations, such as Ca^2+^, forming a water-insoluble gel that is thermo-irreversible. Alginates with a low M/G ratio form rigid gels, while those with a high M/G ratio form elastic gels [27,28,31]. As reported in several studies, alginates exhibit anti-anaphylaxis, immunomodulatory, anti-inflammatory, and antioxidant properties [28]. Alginates reduce blood pressure and cholesterol levels, prevent the absorption of heavy metals in the body, contribute to the prevention of diabetes and adiposity, are active against microorganisms—such as *Escherichia coli* and species of *Staphylococcus*, *Listeria*, and *Salmonella*—and possess anticarcinogenic properties [32,33,34,35,36].

Overall, their adjustable gelling properties, in conjunction with the low cost of their production, render them excellent biomaterials for various applications in the biomedical sector [28,29,36]. Even though the electrospinning of pure alginates is very difficult due to their polyelectrolyte nature, their lack of chain entanglements, the high gelation, and the high surface tension of the spinning solutions, numerous studies for various applications have been conducted with alginate-based electrospun nanofibers [16,22,25,37]. The electrospinnability can be enhanced by chemical modifications that can alter the solubility of alginates in aqueous and/or organic solvents. The high degree of inter- and intramolecular hydrogen bonding between the carboxyl and hydroxyl groups of alginates can be decreased by their replacement with other functional groups [22]. Ku et al. (2014) reported that oxidation of the alginate backbone, resulting in the cleavage of the uronate ring, enhanced the electrospinnability of pure alginate, enabling the production of uniform nanofibers [38]. Furthermore, the sulfation of hydroxyl groups enhanced the solubility of alginate in aqueous solutions and improved its electrospinnability [39]. In a recent study, Sun et al. (2019) performed a graft modification with polyacrylonitrile to enhance the electrospinnability of sodium alginate (SA). Polyacrylonitrile units were grafted on the SA skeleton upon reaction with the alginate hydroxyl groups, thus increasing the alginate’s hydrophobicity. The SA–polyacrylonitrile copolymer was subsequently blended with polyethylene glycol (PEG) in an acetone/DMSO solution, and was electrospun into nanofibers with uniform morphology, enhanced water resistance, and improved thermal properties [40].

Given the hydrophilic character of alginates, a common strategy to improve the mechanical properties and ensure the structural integrity of alginate nanofibers for applications in various biomedical systems is their modification using various crosslinking agents. Divalent cations, such as Ca^2+^, can ionically interact with two carboxyl groups on different alginate chains in an egg-box-like structure, modifying alginate’s hydrophilicity. CaCl_2_ is the most widely applied crosslinking agent for SA-based nanofibers. However, in many in vivo applications, calcium alginate (CA) nanofibers may lose their stability in solutions containing sodium salts, due to the replacement of calcium with sodium. To increase their stability in simulated body fluids, alginate nanofibers can be alternatively crosslinked via covalent bonding of hydroxyl groups with crosslinking agents, such as epichlorohydrin, glutaraldehyde, hexamethylene diisocyanate, and adipic acid hydrazide [41,42]. Another crosslinking approach to alginate nanofibers is through polyelectrolyte complexes with positively charged copolymers, such as chitosan. The ability of alginate to form polyelectrolyte complexes with chitosan via ionic complexation has been demonstrated by Jeong et al. (2011), reporting that alginate and chitosan can crosslink in situ during the electrospinning process to form stable nanofibers suitable for tissue regeneration applications [43]. The spontaneous complexation of the anionic alginate with the cationic chitosan was also investigated by Nista et al. (2015), who obtained nanofibers with a core–shell structure. Through coaxial electrospinning of alginate/PEO (core) and chitosan/PEO (shell), core–shell nanofibrous scaffolds were prepared and, due to the ionic crosslinking between the polysaccharide chains, were able to preserve the nanofibrous structure even after one day of incubation in water [44].

Wound healing is a complex biological process involving inflammation, cell proliferation, and differentiation. Over the years, nanofibrous wound dressing systems have gained significant research interest [22,25,45]. Exhibiting a wide range of desired properties (e.g., biocompatible, non-toxic, highly absorptive, hemostatic), alginates have been extensively explored, in various forms, such as hydrogels, topical formulations, films, foams, and nanofibers, for wound healing applications [45,46]. Alginate dressings absorb wound fluids and form gels that can maintain moisture at the wound area at physiological levels, reducing bacterial activity and promoting the wound recovery process. The therapeutic efficacy of alginate nanofibers can be influenced by the presence of other polymers, crosslinkers, excipients, and antibacterial agents [22,25,37]. Coşkun et al. (2014) developed SA/PVA nanofibers and compared them with commercial wound dressings. The in vivo evaluation, conducted on male New Zealand rabbits, indicated that the nanofibrous patches were able to survive on the wound crust in the early stages of healing, exhibiting the best healing performance, and serving as artificial skin until the regeneration of new tissue [47].

Recently, a multifunctional, trilayer nanofibrous membrane composed of chitosan/PVA/fibrin (lower layer), SA/PVA (middle layer), and gelatin (top layer) was developed for burn wound healing applications. Chitosan and fibrin loaded in the lower layer were used to induce tissue regeneration and blood coagulation. SA incorporated in the middle layer was used to provide enhanced antibacterial protection, and gelatin in the external layer was used to adsorb wound exudates. The fabricated trilayer hybrid membrane exhibited significant antibacterial activity against *Staphylococcus aureus* and *E. coli*, and high absorbent properties, verifying its potential as a wound dressing material [48]. Electrospun alginate nanofibers encapsulating various antimicrobial agents have shown enhanced antibacterial activity, accelerating the healing process [49,50]. Zhu et al. (2016) developed core–shell electrospun nanofibers of chitosan/asiaticoside and alginate/PVA that served as the core and the shell, respectively, to evaluate their effectiveness in the treatment of burn injuries. The scaffolds exhibited high loading capacity and fast release, which facilitated the healing of the wounds. The in vivo study on male Sprague–Dawley rats confirmed the ability of these scaffolds to heal deep partial-thickness burn injuries [51]. Following another approach, Mokhena et al. (2017) prepared alginate nanofibers coated with chitosan/silver nanoparticles. The nanoparticles were well dispersed on the surface of the nanofibers, providing stable polyelectrolyte complexed nanofibers with high antibacterial efficacy against Gram-positive and Gram-negative bacteria [52]. Honey-loaded alginate/PVA electrospun nanofibers were developed by Tang et al. (2019) and tested as wound dressings. By increasing the honey content, the nanofibers exhibited enhanced antioxidant activity, as well as antibacterial activity against *S. aureus* and *E. coli*, while cytotoxicity evaluation confirmed their biocompatibility and their potential as wound dressings [53]. Pakolpakcil et al. (2019) developed pH-sensing halochromic nanofibers via preparation of SA/PVA fiber mats incorporating anthocyanins isolated from black carrot or purple cabbage. The nanofibrous mats successfully displayed color changes upon pH variations that occur during the wound healing process, thus providing an easy way to monitor the progress of healing [54,55].

**Figure 3:**
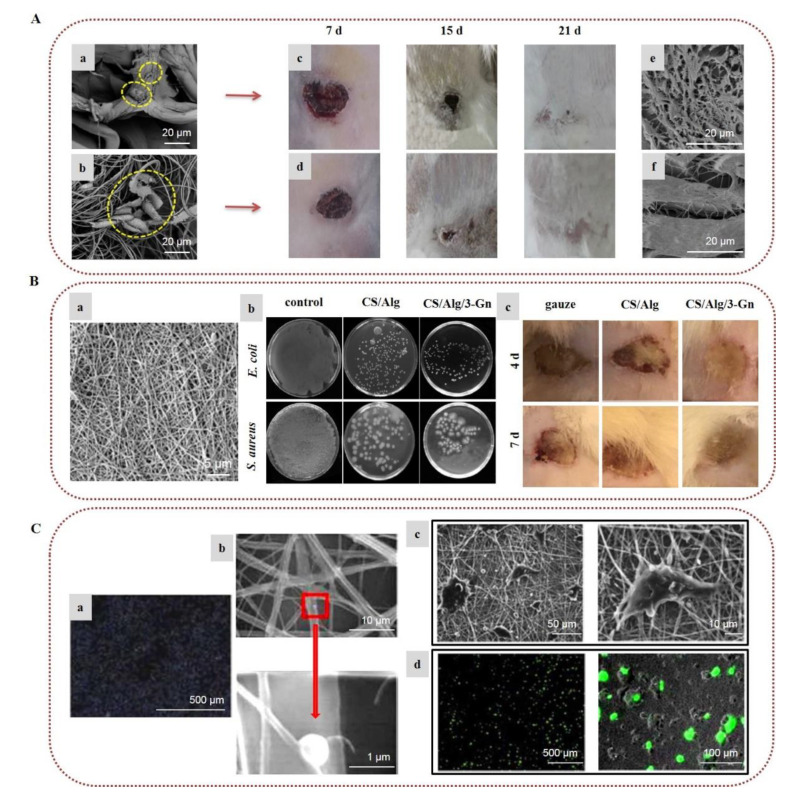
Alginate-based electrospun nanofibers for wound healing applications: (**A**) Attachment and growth of fibroblast cells seeded on (**a**) PVA/SA and (**b**) PVA/SA/spider silk nanofibers. Images of the in vivo wound healing study on rabbit models treated with (**c**) PVA/SA and (**d**) PVA/SA/spider silk nanofibers, on post-treatment days 7, 15, and 21. SEM images of collagen deposition under the wound healing site at 7 days post-treatment with (**e**) PVA/SA and (**f**) PVA/SA/spider silk nanofibers. Reprinted/adapted with permission from [56], Copyright 2021, Elsevier. (**B**) (**a**) SEM image of gentamicin-loaded alginate/chitosan (CS/Alg/3-Gn) nanofibers. (**b**) Antibacterial activity of control, chitosan/alginate (CS/Alg), and CS/Alg/3-Gn against *E. coli* and *S. aureus*. (**c**) Images of the in vivo burn wound healing study on murine models treated with gauze, CS/Alg, or CS/Alg/3-Gn on days 4 and 7. Reprinted/adapted with permission from [63], Copyright 2020, Elsevier. (**C**) (**a**) Gene immobilization onto alginate/PCL composite nanofibers. To determine gene immobilization, DNA was stained with Hoechst 33258 before complexing with polyethylenimine. (**b**) SEM images of non-viral vectors’ adsorption onto alginate/PCL nanofibers. (**c**) SEM images of HEK-293T cells cultured on alginate/PCL nanofibers for 3 days. (**d**) In situ transfection study on nanofibers. Fluorescent images of GFP expression from transfected HEK-293T cells cultured on alginate/PCL nanofibers immobilized with DNA/polyethylenimine nanoparticles for 3 days. Reprinted/adapted with permission from [70], Copyright 2018, Elsevier.

In a recent investigation, natural spider silk was embedded into SA/PVA nanofibrous scaffolds. In vivo tests on rabbit models and in vitro biocompatibility with cellular behavior assays on murine fibroblast cells (L929) indicated that the nanofibrous mat loaded with the natural spider silk induced rapid healing of skin scars by decreasing the numbers of inflammatory cells and improving the collagen formation rate and proliferative cell activity (Figure 3A) [56]. In a recent work, ZnO nanoparticles, acting as an antimicrobial agent, were encapsulated in alginate/PVA electrospun nanofibers, providing remarkable antimicrobial activity against *E. coli*, *S. aureus*, and *Candida albicans* [57]. In a similar manner, Dodero et al. (2020) fabricated a multilayer electrospun scaffold composed of PCL and crosslinked alginate-incorporating ZnO nanoparticles, using PEO as the co-spinning polymer of alginate. A washing/crosslinking process was applied using EtOH–SrCl_2_ to remove PEO and stabilize the nanofibrous structure. The external hydrophobic PCL layer provided liquid-repellent abilities, good mechanical properties, and protection from the outer environment. The internal alginate layer promoted tissue regeneration, while ZnO nanoparticles provided strong antibacterial activity, indicating that the fabricated scaffolds can lead to promising wound healing patches [58].

The incorporation of various APIs in alginate nanofibers is a popular and efficient approach for the development of wound dressings with enhanced wound healing properties [59,60,61,62,63,64,65]. Bakhsheshi-Rad et al. (2020) designed SA/chitosan nanofibers loaded with gentamicin, and evaluated their healing efficacy on wound burns. Antimicrobial activity tests showed that the composite fibers were highly efficient against *S. aureus* and *E. coli*. The conducted in vivo study on a BALB/C mouse model demonstrated that the nanocomposites accelerated wound healing, providing increased re-epithelialization, collagen deposition, and development of blood vessels and hair follicles (Figure 3B) [63]. In another recent study, electrospun nanofibers of alginate/PVA/gelatin encapsulating ciprofloxacin were developed and tested for their antimicrobial activity. The chemical integrity of ciprofloxacin after the electrospinning process was confirmed by ^1^H NMR spectroscopy, while the release rate studies showed that the release of ciprofloxacin from the fiber mats was decelerated with the increase in gelatin content. The nanofibers exhibited significant antibacterial properties against *S. aureus* and *E. coli*, and adequate mechanical properties, indicating their potential utilization as wound dressings [64].

Electrospun alginate nanofibers have also been widely used for the development of novel drug delivery systems for the targeted or controlled release of various APIs [22,37]. Qi et al. (2006) fabricated composite nanofibers blending CA microspheres with the hydrophobic poly(l-lactic acid) (PLLA). The fibers were fabricated via emulsion electrospinning and revealed a unique beads-in-string structure, with the CA microspheres serving as reservoirs for the encapsulation of the hydrophilic bovine serum albumin (BSA). This microencapsulation technique proved effective for the encapsulation of hydrophilic agents, such as BSA, to achieve prolonged release profiles and low burst-release rates [66]. Pegg et al. (2014) prepared ammonium alginate derivatives via ionic linkage with amine-containing drugs (e.g., lidocaine, neomycin, papain) and electrospun them in blends with PVA. The resulting nanofibers were able to release the drugs while retaining their function, with great reproducibility [67]. In another study, amphiphilic derivatives of alginate were synthesized by grafting octyl amines onto the carboxylic groups of alginate, and were successfully electrospun with PVA, incorporating the hydrophobic drug λ-cyhalothrin. The release profile of these nanofibers was comparatively studied against SA/PVA nanofibers that were also loaded with λ-cyhalothrin. The amphiphilic derivatives of alginate/PVA nanofibers presented a slow and prolonged release of the drug, while the SA/PVA fibers exhibited a burst release, indicating that the amphiphilic derivatives of alginate could be further explored as carriers of hydrophobic drugs [68]. Tamizi et al. (2018) prepared core–shell nanofibrous structures of SA/PVA for the sustained delivery of dexpanthenol. SA/PVA/dexpanthenol served as the core and PVA/ammonium peroxydisulfate as the shell layer, with ammonium peroxydisulfate added as a stabilizer to improve the mechanical properties of the nanofibers and reduce their degradation rate. The release profile study indicated that the scaffolds were able to release dexpanthenol for a sustained period [69].

Hu et al. (2018) designed alginate/PCL nanofibers with immobilized polyethylenimine/DNA complexes for the in situ transfection of genes for gene delivery applications. Due to the non-adhesive properties of alginate, PCL was incorporated in the scaffold to promote cell adhesion and improve the biocompatibility of the scaffold, whereas the immobilization of the genes was accomplished due to the anionic character of alginate. Alginate adsorbed the cationic polyethylenimine/DNA complexes, with higher alginate content resulting in increased immobilization of non-viral vectors. By optimizing the ratio of PCL and alginate, the in situ transfection study verified that the composite nanofibers delivered target genes to the adhered cells and improved cell viability (Figure 3C) [70,71]. Wen et al. (2018) prepared a core–sheath nanofibrous colon-specific delivery system of quercetin and prebiotics with enhanced anticancer properties. SA/PEO was used as a shell layer due to the ability of alginate to resist the acidic conditions in the gastric tract, forming a viscous gel. Quercetin-loaded chitosan nanoparticles/PVA incorporating prebiotics were used as a core layer, as chitosan does not dissolve in the small intestine, and can be degraded by microbial enzymes in the colon. The release profile study in gastrointestinal-like fluids indicated a sustained and colon-targeted release of quercetin, whereas the release rate was enhanced by the incorporation of prebiotics. It is worth noting that the fiber mats caused a significant decrease in colon cancer cells’ growth, indicating that the fabricated scaffolds could be utilized for the oral therapy of colon cancer [72]. In another study, bilayer nanofibrous scaffolds of PVA/SA and PEO were prepared and loaded with different drugs in order to combine strong and mild painkillers for the pain management of burns. The wound contact layer consisted of PEO loaded with gabapentin (a strong nerve painkiller), covered by a layer of PVA/SA nanofibers loaded with acetaminophen (a mild painkiller). The PEO nanofibrous layer showed a burst release of gabapentin, while the SA in the PVA/SA nanofibers was converted to CA by ionic crosslinking with CaCl_2_ in order to achieve slow release of the encapsulated acetaminophen. Such fabricated bilayer drug-loaded nanofibers with the dual release function of strong and mild painkillers could serve as a promising approach to effectively handle the pain of burns with fewer side effects [73]. Recently, Mirzaie et al. (2021) prepared SA/PVA/graphene oxide nanofibers and film matrices loaded with curcumin and studied their performance as drug carriers. For both fibers and film, a reduced drug release and higher levels of anticancer activity against MCF-7 cells were observed when increasing the amounts of graphene oxide and curcumin, respectively. However, the nanofibers showed higher drug loading and lower drug release rates compared to the film, suggesting that SA/PVA/graphene oxide nanofibers are a promising carrier of curcumin [74].

**Figure 4:**
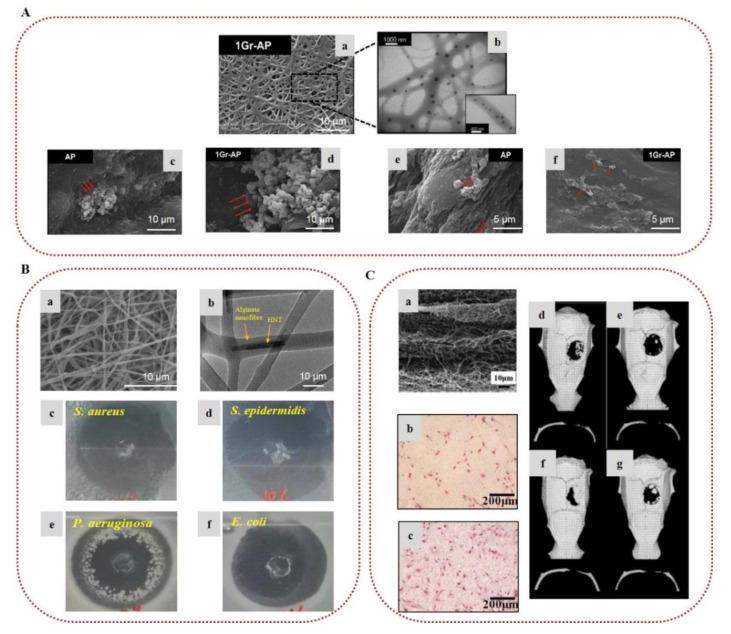
Alginate-based electrospun nanofibers for tissue engineering applications: (**A**) (**a**) SEM image of SA/PVA nanofibers incorporating graphene nanosheets (Gr-AP). (**b**) TEM image of the Gr-AP scaffold. SEM images of PC12 cell attachment on (**c**) alginate/PVA (AP) and (**d**) Gr-AP nanofibers after 1 day. Spreading of cells on (**e**) AP and (**f**) Gr-AP nanofibers after 7 days. Reprinted/adapted with permission from [76], Copyright 2017, Elsevier. (**Β**) (**a**) SEM image of alginate/PVA nanofibers incorporating cephalexin-loaded halloysite nanotubes. (**b**) TEM image showing halloysite nanotubes on alginate/PVA nanofibers. Antibacterial activity of alginate-based scaffolds against (**c**) *S. aureus,* (**d**) *S. epidermidis*, (**e**) *P. aeruginosa*, and (**f**) *E. coli*. Reprinted/adapted with permission from [77], Copyright 2018, American Chemical Society. (**C**) (**a**) Microstructure of a composite membrane with 5 repeated units of hydrophobic PLA/nano-hydroxyapatite and hydrophilic PVA/SA/nano-hydroxyapatite layers in a sandwich-like structure. Images of alkaline phosphatase activity of BMSCs cultured on (**b**) a single PLA/nano-hydroxyapatite layer and (**c**) a composite membrane of 5 units after 7 days of culture on the membranes. Computed tomography images of rat skulls: (**d**) composite membrane group 3 weeks after operation, (**e**) blank group 3 weeks after operation, (**f**) composite membrane group 6 weeks after operation, and (**g**) blank group 6 weeks after operation. Reprinted/adapted with permission from [83], Copyright 2021, Elsevier.

In tissue engineering, cells, scaffolds, and bioactive molecules interact to restore or maintain various biological tissues. Composite electrospun alginate-based nanofibers have been broadly investigated as biomimetic scaffolds to support cell adhesion and provide appropriate mechanical support for various tissue engineering applications, including nerve, skin, and bone regeneration [22,25,37]. To overcome the inherent non-adhesive properties of alginate, Jeong et al. (2010) modified alginate by covalently bonding a cell-adhesive peptide (GRGDSP) and electrospun it with PEO. The presence of the peptide enhanced the attachment, spread, and proliferation of primary human dermal fibroblast (HDF) cells on the surface of the nanofibers [75]. In order to mimic the ECM of the peripheral nerve, Golafshan et al. (2017) developed SA/PVA nanofibers loaded with graphene nanosheets. The hybrid scaffolds displayed superior mechanical properties and electrical conductivity, with enhanced attachment, spread, and proliferation of PC12 cells, indicating the great potential of such hybrid graphene–alginate scaffolds for peripheral nerve regeneration (Figure 4A) [76]. Silva et al. (2018) studied the incorporation of cephalexin-loaded halloysite nanotubes into alginate/PVA nanofibers via electrospinning for tissue regeneration applications. The electrospun alginate-based nanofibrous scaffolds possessed robust mechanical properties and sustained antimicrobial protection against *S. aureus*, *Staphylococcus epidermidis, Pseudomonas aeruginosa*, and *E. coli*, showing great potential for their use as an artificial ECM scaffold (Figure 4B) [77]. Lee et al. (2020) prepared 3D hybrid alginate scaffolds composed of alginate nanofibers layered on 3D-printed microscale alginate struts encapsulating human adipose stem cell spheroids to stimulate angiogenesis and induce skin repair. The entrapped spheroids retained their function, and were able to express angiogenic and wound healing-related genes, suggesting that such hybrid scaffolds could provide new insight into skin tissue regeneration [78].

In the field of bone tissue engineering, Chae et al. (2013) encapsulated hydroxyapatite in alginate-based nanofibers via in situ synthesis of hydroxyapatite nanocrystals on the surface of the nanofibers during crosslinking with Ca^2+^ ions, which led to uniform mineralization. The presence of hydroxyapatite improved the mechanical properties of the nanofibers and provided stable attachment, stretching, and elongation of rat calvarial osteoblast (RCO) cells, as well as a high proliferation rate, indicating that such alginate nanocomposites could serve as promising candidates for bone regeneration applications [79,80]. Cesur et al. (2019) developed electrospun nanofibers of SA/PLA loaded with tricalcium phosphate derived from orange spiny oyster shells. The fiber mats exhibited advanced mechanical properties due to the presence of SA and tricalcium phosphate. They also demonstrated adequate cytocompatibility and high cell viability of SaOS-2 cells [81]. Ye et al. (2019) fabricated nanofibrous scaffolds of CA and PLA for the regeneration of periodontal bone tissue. The presence of CA improved the mechanical strength of the scaffold and provided adequate space for tissue regeneration, enhancing the hydrophilicity of the fibers. The conducted in vitro assay revealed that the scaffolds promoted cell adhesion of periodontal ligament cells and induced osteogenic differentiation of bone marrow mesenchymal stem cells (BMSCs) into osteoblasts. Furthermore, the incorporation of CA led to an increase in the number of mineralized nodules by increasing the expression of mineralized genes, indicating that the CA nanofibers represent a promising scaffold for the tissue repair of periodontal bone [82]. In a recent study, electrospun nanofibrous membranes with repeated hydrophobic PLA/nano-hydroxyapatite (A) and hydrophilic PVA/SA/nano-hydroxyapatite (B) layers in a sandwich-like structure (ABA, BAB) were successfully prepared for skull defect repair. By increasing the number of AB units, the cohesion and the mechanical properties of the electrospun membranes were improved due to the enhanced entanglement of the hydrophobic and hydrophilic fibers. Furthermore, the composite membranes exhibited better biological activity, as compared to a single layer of PLA/nano-hydroxyapatite, enhancing the differentiation of BMSCs in vitro and accelerating the skull defect repair in vivo after subcutaneous implantation in the dorsomedial skin of male Sprague–Dawley rats (Figure 4C) [83].

Irani et al. (2021) developed electrospun fiber mats of alginate sulfate and PVA for cartilage tissue engineering. Alginate sulfate was selected as an efficient substitute for bioactive glycosaminoglycans (GAGs) to be used as a functional support system. The produced nanofibers showed appropriate cytocompatibility and promoted bone marrow mesenchymal stem cells’ growth and differentiation into chondrocytes, demonstrating the great potential of alginate sulfate nanofibers for cartilage tissue engineering applications [84]. Due to the anti-adhesive properties of alginates, novel anti-adhesion barriers based on alginate electrospun nanofibers have been reported to prevent postoperative peritoneal adhesion [85,86]. Specifically, Chang et al. (2012) demonstrated the anti-adhesion ability of alginate/chitosan nanofibers by utilizing alginate and chitosan in a core–sheath fiber structure. In vitro cytocompatibility tests revealed that alginate reduced cell attachment and protein adsorption, whereas chitosan inhibited proliferation and triggered apoptosis. The in vivo evaluation on female Wistar rats demonstrated that the developed alginate–chitosan nanofibers were highly effective in reducing peritoneal adhesions, and could serve efficiently as a tissue anti-adhesion barrier [85].

### 2.2. Carrageenans

Carrageenans include a family of sulfated anionic galactans formed by alternate units of d-galactose and 3,6-anhydro-galactose linked by α-1,3 and β-1,4 glycosidic bonds. They are the main component of the cell walls of red seaweeds of the class Rhodophyceae [87]. Carrageenans, with molecular weights varying from 100 to 1000 kDa, are classified according to the degree of sulfation and the content of 3,6-anhydro-galactose into three main groups, namely, kappa (κ)-carrageenans (one sulfate group and one 3,6-anhydro-galactose per disaccharide), iota (ι)-carrageenans (two sulfate groups and one 3,6-anhydro-galactose per disaccharide), and lambda (λ)-carrageenans (three sulfate groups and no 3,6-anhydro-galactose per disaccharide) [88]. The sulfate content in κ- and ι-carrageenans is similar, and accounts for approximately 25–30%, while in λ-carrageenans the sulfate substitution is higher, accounting for around 32–39% [30,89]. However, seaweeds do not always produce these idealized carrageenans, often instead producing hybrid structures of κ/ι-carrageenans that contain repeating units of both κ- and ι- carrageenans [89]. All carrageenan types are soluble in water, but only κ- and ι-carrageenans form gels in the presence of cations (typically K^+^ or Ca^2+^), while λ-carrageenans do not form gels, due to their high sulfate content. Gelation of carrageenans is thermo-reversible, and is affected by the nature and the concentration of the cations, as well as the concentration of the polysaccharide [1,87,89]. Many biological properties of carrageenans have been reported in the literature, including anticoagulant, antiviral, antitumor, immunomodulatory, antioxidant, and anti-hyperlipidemic activities [6,8,87,88,89]. Due to their physicochemical and biological properties, carrageenans have been widely exploited in the food, cosmetics, and pharmaceutical industries [88,89].

Aqueous solutions of carrageenans are generally highly viscous and difficult to electrospin [90]. As a result, there are only a few reports in the literature describing carrageenan electrospun nanofibers. Goonoo et al. (2017) developed electrospun scaffolds of κ-carrageenan blended with polyhydroxybutyrate or polyhydroxybutyratevalerate (PHBV) in 1,1,1,3,3,3-hexafluoro-2-propanol (HFIP)/CHCl_3_. The in vitro study showed that the incorporation of κ-carrageenan led to the generation of apatite crystals with nanoscale dimensions on the surface of the fibers, and improved the proliferation of NIH3T3 cells. In contrast to pure polyester fibers, carrageenan fiber mats enhanced the osteogenic differentiation and mineralization of human osteosarcoma cells (SaOS-2), highlighting the potential of the fabricated scaffolds for bone tissue engineering applications [91]. In a recent study, ι-carrageenan/PVA electrospun nanofibers loaded with partially reduced graphene oxide were successfully fabricated for wound healing and skin repair applications. Antimicrobial activity evaluation revealed that the fiber mats provided higher antimicrobial activity against *E. coli*, *Bacillus* sp., and *Staphylococcus* sp., as compared to ampicillin and tetracycline. Moreover, the in vivo study employing male Wistar rats with infected (by *E. coli* and *Staphylococcus* sp.) and non-infected wounds demonstrated their efficacy in accelerating the healing process and promoting hair follicles’ growth in the presence of yeast [92].

It has been reported that carboxymethylation of κ-carrageenans, affording structural analogues of heparin, may improve their properties, lowering the solution viscosity and enhancing the antibacterial and anticoagulant activities [90,93]. Along these lines, Madruga et al. (2020) investigated the development of carboxymethyl-κ-carrageenan/PVA electrospun nanofibers as a biomaterial for tissue engineering applications. The nanofibers were thermally crosslinked and remained stable in water for 48 h. The incorporation of carboxymethyl-κ-carrageenan enhanced the cell adhesion and proliferation of human adipose-derived stem cells, and promoted osteogenic differentiation in vitro, while at the same time improving the biodegradability and cytocompatibility of the fiber mats [90]. Furthermore, Madruga et al. (2021) demonstrated that carboxymethyl-κ-carrageenan/PVA electrospun nanofibers promoted blood coagulation activity and exhibited significant antibacterial activity against *S. aureus* and *P. aeruginosa*, showing their great potential as wound dressing materials [94]. In another study, Abou-Okeil et al. (2021) fabricated uniform nanofibers of oxidized κ-carrageenan blended with hyaluronic acid/PVA. The produced nanofibers exhibited significant antibacterial activity against Gram-positive (*S. aureus*) and Gram-negative (*E. coli*) bacteria [95].

### 2.3. Ulvans

Ulvans are a group of anionic sulfated polysaccharides found in the cell walls of green algae, mainly of the genera *Ulva* and *Enteromorpha*. Accounting for 9–45% of the dry weight of the seaweed biomass, the chemical composition of ulvans displays great heterogeneity [96]. Ulvans are mainly composed of sulfated rhamnose, xylose, and iduronic and glucuronic acids arranged in α- and β-1,4-linked units. Glucuronic or iduronic acid and rhamnose in the form of aldobiuronic acids comprise the main disaccharide units of ulvans, denoted as type A and B ulvanobiuronic acids. Ulvanobiuronic acid type A3s consists of β-d-glucuronic acid 1,4-linked to α-l-rhamnose 3-sulfate, while in type B3s α-l-iduronic acid is 1,4-linked to α-l-rhamnose 3-sulfate [97,98]. The exact composition of ulvans, ranging in molecular weight between 150 and 2000 kDa, varies according to the seaweed species, the area of collection, the harvesting period, and post-collection processing [99]. Several studies have reported that ulvans exhibit a wide range of biological properties, including antioxidant, anticoagulant, antiviral, anti-hyperlipidemic and immunomodulatory activity [96,100,101], which are influenced by the sugar composition, the molecular weight, and the sulfate content [97,98]. In addition, a number of studies have demonstrated the ability of ulvans to promote the attachment and proliferation of osteoblasts [96,98]. Due to their chemical resemblance to GAGs, their gelling properties and bioactivities, ulvans have shown potential for many biomedical applications, such as tissue engineering and drug delivery systems.

**Figure 5:**
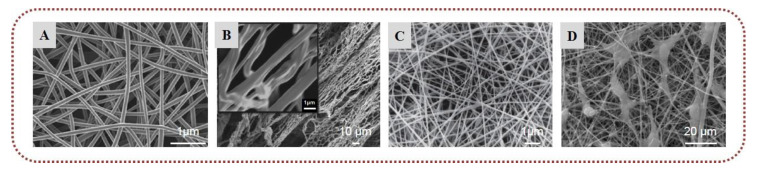
Ulvan-based nanofibers: SEM images of (**A**) ulvan/PVA (1:1). Reprinted/adapted with permission from [102], Copyright 2011, Elsevier; (**B**) ulvan/PCL (3:8). Reprinted/adapted with permission from [103], Copyright 2015, John Wiley and Sons; (**C**) ulvan/PVA (2:1). Reprinted/adapted with permission from [104], Copyright 2021, Elsevier; (**D**) fibroblasts adopting a stellate morphology on ulvan cellulose acetate/polydioxanone fibers. Reprinted/adapted with permission from [105], Copyright 2021, Elsevier.

To date, there have only been a few reports concerning the incorporation of ulvans into electrospun nanofibers. Toskas et al. (2011) developed for the first time electrospun nanofibrous matrices of ulvan blended with PVA at different ratios in the presence of calcium ions and boric acid. The nanofibers were collected as a nonwoven membrane without interconnections, exhibiting a high degree of orientation attributed to the presence of ulvan (Figure 5) [102]. Subsequently, the preparation of ulvan-based nanofibers has been reported from blends of ulvan with either PCL or PEO at various concentrations. SEM analysis revealed that the average diameter of the nanofibers decreased when the ulvan content increased, with the nanofibers exhibiting a spider-web-like network in the case of ulvan/PCL blends and a spindle-like morphology for ulvan/PEO blends. The composite nanofibers exhibited good compatibility and strong interaction between ulvan and the two copolymers, suggesting that such hybrid structures could lead to the development of novel biomaterials with synergetic functional properties of the combined ingredients [103].

Mandany et al. (2021) investigated the preparation of ulvan/PVA nanofibers from ulvan extracted from micro- or nano-powdered algae with or without organic solvent pretreatment. By using deionized water as a solvent, bead-free fibers were obtained from ulvan samples of non-pretreated algal micropowder, most probably due to their higher molecular weight, whereas intermolecular interactions resulted in bead-free nanofibers with strong mechanical properties for both pretreated and non-pretreated algal nanopowder samples [104]. Madub et al. (2021) obtained an extract of cellulose and ulvan from green seaweeds that underwent chemical modification for the formation of cellulose acetate, and subsequently fabricated electrospun nanofibrous mats with or without PLA and polydioxanone as copolymers. The ulvan/cellulose-based nanofibers showed accelerated growth of fibroblast cells in vitro, most probably attributable to the presence of ulvan, which displays similarity to mammalian GAGs of the native ECM. Moreover, enhanced angiogenesis with low foreign body response was observed in male and female Wistar rats, indicating the good tissue–material interaction of the ulvan/cellulose scaffolds [105].

### 2.4. Fucoidans

Fucoidans represent a group of sulfated polysaccharides present in the cell walls of brown seaweeds (accounting for 10–20% of the total dry weight), and in some echinoderms (i.e., sea cucumbers and sea urchins) [97]. They are highly heterogeneous polysaccharides, mainly composed of sulfated l-fucose, but they also contain small proportions of uronic acid, galactose, arabinose, mannose, xylose, and glucose [30,106]. Their general backbone consists of α-1,3-fucopyranose residues or alternating α-1,3- and α-1,4-linked fucopyranosyls [107]. Acetate or sulfate substitutions and/or side branches bearing fucopyranoses or other glycosyl units may also be found in their backbone [108]. The chemical composition of fucoidans is related to the organism, the harvest season, and the extraction method [109]. Fucoidans isolated from seaweeds are highly branched, while those from echinoderms are linear [110]. The physicochemical characteristics and biological properties of fucoidans are strongly related to their chemical structure. Specifically, the sulfate content, as well as the degree of branching, determines the solubility of fucoidans. Moreover, the position and the content of the sulfate groups affect their biological characteristics [30,111]. In recent years, fucoidans have been broadly studied because of their numerous biological properties, which include anticoagulant, antiviral, antitumor, immunomodulatory, antioxidant, anti-hyperlipidemic, and anti-inflammatory activities [111,112].

Electrospun nanofibers based on fucoidans have been successfully developed for various biomedical applications. Lee et al. (2012) designed nanofibers of fucoidan blended with PCL for bone tissue regeneration applications, using CH_2_Cl_2_/DMF as the electrospinning solvent system. In order to examine the influence of fucoidan on cellular response, MG63 osteoblast-like cells were cultured on these biocomposites. SEM analysis revealed that the cells were widely distributed on the surface of the fibers, and were agglomerated when the concentration of fucoidan increased. Furthermore, the incorporation of fucoidan in the fiber mats led to higher alkaline phosphatase activity and calcium mineralization in comparison to those observed for neat PCL nanofibers [113]. Rujitanaroj et al. (2014) fabricated electrospun fucoidan, pullulan, and dextran nanofibers loaded with vascular endothelial growth factor (VEGF) for vascular tissue engineering applications. The nanofibers were crosslinked in situ during the electrospinning process using trisodium trimetaphosphate, which interacted with the hydroxyl groups of fucoidan, allowing for an increased fucoidan content in the fiber mats. The incorporation of fucoidan resulted in a sustained release of VEGF, and prolonged bioactivity due to the interactions between the sulfate groups and VEGF. In vivo studies on mice showed that the fiber mats displayed improved vascularization response and cellular infiltration, suggesting that fucoidan could act as a customized container for the effective delivery of VEGF [114].

**Figure 6:**
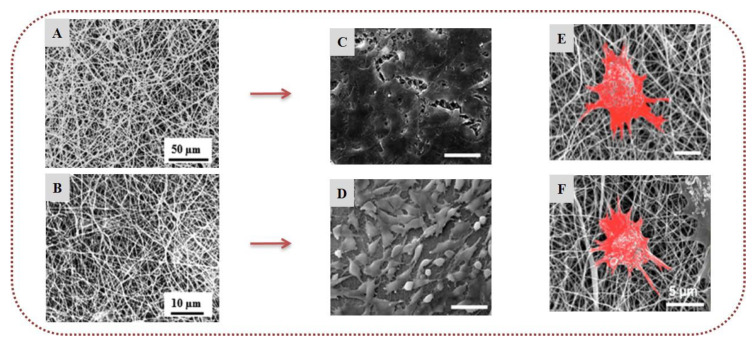
Fucoidan and κ-carrageenan-based nanofibers: SEM images of (**A**) polydioxanone/κ-carrageenan (70/30) electrospun nanofibers and (**B**) polydioxanone/fucoidan (70/30) electrospun nanofibers. NIH3T3 cells seeded on (**C**) polydioxanone/κ-carrageenan and (**D**) polydioxanone/fucoidan nanofibers after 7 days. SaOS-2 cells seeded on (**E**) polydioxanone/κ-carrageenan and (**F**) polydioxanone/fucoidan nanofibers in osteogenic differentiation medium. Reprinted/adapted with permission from [116], Copyright 2017, American Chemical Society.

In another study, electrospun scaffolds of fucoidan, chitosan, and PVA were developed successfully and crosslinked with glutaraldehyde to prevent their dissolution in water. SEM analysis revealed that the nanofibers were uniform, with an interconnected pore structure similar to the ECM. The release profile study using BSA as a drug model revealed that fucoidan-based fiber mats exhibited a decelerated release as compared to that of chitosan/PVA nanofibers. Moreover, the incorporation of fucoidan in the fiber mats enhanced the anticoagulant and antithrombotic activities of the scaffolds, indicating the high potential of fucoidan for vascular tissue engineering applications [115]. Goonoo et al. (2017) developed electrospun nanofibers of polydioxanone blended with fucoidan or κ-carrageenan for tissue engineering applications. The incorporation of the polysaccharides enhanced the viability of fibroblast cells (NIH3T3) and human osteosarcoma cells (SaOS-2). Polydioxanone/fucoidan nanofibers induced greater osteogenic differentiation of SaOS-2 cells, whereas polydioxanone/κ-carrageenan fibers showed higher proliferation of NIH3T3 cells, highlighting the potential of both fucoidan and κ-carrageenan for bone tissue engineering applications (Figure 6) [116]. In a recent study, chitosan-modified electrospun nanofibers of fucoidan/ultrahigh-molecular-weight PEO promoted the adhesion of vascular endothelial cells (HUVECs) and inhibited the adhesion of monocytes, demonstrating that they could serve as promising vascular tissue engineering scaffolds [117].

### 2.5. Agar

Agar, isolated from red seaweeds, is a mixture of two polysaccharides, namely agaroses and agaropectins. Agaroses are linear polysaccharides composed of d-galactose and 3,6-anhydro-l-galactose units linked by β-1,3 and α-1,4 glycosidic bonds that constitute up to 70% of agar [118]. The structure of agaropectins is more complicated, since it consists of d-galactose, 3,6-anhydro-l-galactose, and residues of sulfonic, pyruvic, and uronic acids, with poor gelling properties. Agar can form a thermo-reversible gel that becomes liquid above 80 °C. The gelling properties of agar are due to the double-helical structure of agarose, which aggregates to make a 3D network that can hold water. It is widely used in the food industry, and has also found applications in microbiology and the pharmaceutical sector [30].

Electrospun nanofibers based on agar have been successfully designed for various applications [119]. The first fabrication of agarose-based electrospun fibers was reported by Teng et al. (2009), who electrospun agarose blended with chitosan in TFA/CH_2_Cl_2_. With agarose and chitosan showing good compatibility and strong interaction, the nanofibers were uniform, with a diameter that decreased when increasing the content of agarose [120]. In another study, a drug delivery system of hybrid ampicillin-loaded agar/polyacrylonitrile electrospun nanofibers was developed, exhibiting a prolonged release of ampicillin and high antibacterial activity against *E. coli* [121]. Sousa et al. (2015) reported that the deep eutectic solvent (2-hydroxyethyl)trimethylammonium chloride/urea facilitated the electrospinnability of agar blended with PVA, allowing for the development of composite microfibers with higher agar content compared to agar-in-water fibers [122]. Xu et al. (2018) fabricated electrospun nanofibers of agarose acetate, blended them with β-tricalcium phosphate, and studied their effectiveness for bone tissue engineering. The encapsulation of β-tricalcium phosphate enhanced the hydrophilicity and mechanical properties of the nanofibers. The scaffolds induced the proliferation and differentiation of rat bone marrow-derived mesenchymal stem cells in vitro, without causing any inflammatory response in vivo, suggesting that such agarose acetate-based fibrous scaffolds could be utilized for bone tissue repair applications [123].

### 2.6. Laminarins

Laminarins are a group of storage polysaccharides with a molecular weight of approximately 5000 Da, naturally occurring in the cell walls of brown algae [124]. They comprise β-glucans linked through β-1,3 glycosidic bonds with some 6-*O*-branching and β-1,6 intrachain links, and display heterogeneity according to the harvest season, environmental factors, and the algal species. There are two types of laminarin chains that differ at the reducing end, which terminates either with d-mannitol (M type) or d-glucose (G type) units [124,125,126]. The level of branching influences the water solubility of laminarins. Various biological functions of laminarins have been reported in the literature, including anti-inflammatory, antioxidant, and anticancer activities [127].

Laminarins are relatively underutilized polysaccharides [30,126], and there are only a few reports concerning laminarin-based electrospun nanofibers. Jung et al. (2012) developed electrospun nanofibers of PCL blended with a brown seaweed extract that contained laminarin and fucoidan to test their effectiveness for the treatment of central nervous system injuries. The fabricated scaffolds were able to increase or decrease astrocyte viability depending on the concentration of the extract. Astrocyte cells are of critical importance in neural regeneration, and such scaffolds could be used to control the activity of astrocytes by adjusting the concentration of the polysaccharides [128]. Kim and Kim (2013) demonstrated that composite fiber mats of PHBV/gelatin/laminarin promoted the proliferation rate of fibroblasts, indicating their potential for tissue engineering [129]. Subsequently, the same group prepared nanofibrous scaffolds of PHBV blended with laminarin or depolymerized laminarin as wound dressings for the treatment of melanoma. Depolymerized laminarin nanofibers enhanced the proliferation rate of fibroblast cells due to their increased antioxidant activity as compared to laminarin, while at the same time more effectively suppressing the proliferation of human malignant melanoma cells [130].

## 3. Marine Animal-Derived Biopolymers

### 3.1. Chitin and Chitosan

Among marine animal polysaccharides, chitin found in the exoskeletons of crustaceans, such as crabs and shrimps, represents one of the most abundant marine biopolymers, with various applications in the biomedical field [3]. This is a group of linear polysaccharides consisting of β-1,4-linked *N*-acetyl-2-amino-2-deoxy-β-d-glucopyranose (*N*-acetyl-d-glucosamine), found in either α-, β-, or γ- form, and is commercially extracted by acid treatment from crab and shrimp shells that occur as food industry waste products [131]. Chitin is insoluble in water; thus, it is usually converted to its deacetylated derivative, known as chitosan, which is soluble in acidic conditions [1]. Chitosan, consisting of *N*-acetyl-d-glucosamine and glucosamine at variable ratios, is naturally found only in some fungi [1,2,3]; therefore, commercial chitosan is obtained as a chitin derivative via chemical or enzymatic deacetylation of chitin. The deacetylation is usually partial, resulting in polymers with a copolymeric structure and various molecular weights, ranging between 300 and 1000 kDa. The differences of the final products in the deacetylation degree and molecular weight impact the physicochemical and biological properties of chitosan and its derivatives. Under acidic conditions, chitosan acts as a polycation, and can form hydrogels with polyanions and polyelectrolytes [132]. Chitin and chitosan, possessing biological properties such as anticoagulant, antimicrobial, antitumor, and anti-inflammatory activities, have been widely investigated for the development of novel biomaterials, especially in the areas of wound healing, drug delivery, and tissue engineering [1,2,133,134].

Electrospinning of chitin is very difficult because it does not dissolve in the most commonly used solvents. The poor solubility of chitin dictates the use of toxic and corrosive solvents, a fact that limits the development and application of chitin nanofibers [135]. Nonetheless, many researchers have investigated the fabrication of chitin electrospun nonwovens. Chitin electrospun nanofibers were successfully generated by Noh et al. (2006) from HFIP solution. To enhance the solubility of chitin in HFIP, chitin was exposed to ^60^Co γ-irradiation to reduce its molecular weight. In cytocompatibility assays, the produced chitin nanofibers induced the adhesion and spread of normal human keratinocytes and fibroblasts, indicating their potential for wound healing and skin regeneration applications [136]. In another study, Kim et al. (2012) developed electrospun nanofibers of chitin blended with PCL in HFIP/TFA. Compared to the neat PCL fibers, chitin-based fibers exhibited improved mechanical properties and high viability of human dermal fibroblasts [137]. Jung et al. (2018) developed electrospun nanofibers of β-chitin from cuttlefish bone blended with PEO in formic acid for application as wound dressings. The fiber mats were immersed in water to remove the PEO and yield nanofibers of pure β-chitin. The performed in vivo study on male Sprague–Dawley rats indicated that both β-chitin/PEO and β-chitin nanofibers prevented inflammatory response, and enhanced epithelial regeneration and collagen deposition, with chitin nanofibers displaying better wound healing efficacy than β-chitin/PEO nanofibers [138]. Recently, Cheng et al. (2021) developed a composite scaffold that was reinforced by silk fibroin/chitin electrospun nanofibers and impregnated with the transforming growth factor-β1 (TGF-β1) to simulate the ECM and promote cartilage regeneration. The incorporation of chitin-based nanofibers enhanced the hardness and elasticity of the scaffold, and promoted the adhesion and proliferation of chondrocytes in vitro, which are of paramount importance for cartilage implants. Furthermore, in vivo experiments on male Sprague–Dawley rats demonstrated that the encapsulation of TGF-β1 significantly accelerated the repair of cartilage defects [139].

To avoid the use of toxic solvents, Shalumon et al. (2009) prepared carboxymethyl chitin, which is a water-soluble derivative of chitin, and electrospun it successfully with PVA. Carboxymethyl chitin/PVA electrospun nanofibers were crosslinked with glutaraldehyde to enhance their resistance in water for up to 48 h. Mineralization studies showed that the fiber mats induced the formation of hydroxyapatite. Additionally, they promoted the adhesion and spread of human mesenchymal stem cells, indicating their potential for tissue engineering applications [140]. In another study, dibutyryl chitin was prepared by adding butyric groups at C-3 and C-6 of chitin, and was successfully electrospun with PLA. The fabricated nanofibers exhibited superior wound healing efficacy in vivo on hairless mice due to the presence of dibutyryl chitin, demonstrating the great potential of dibutyryl chitin for wound treatment [141]. Following another approach, chitin whiskers were grafted with l-lactide (g-CHWs), and were successfully electrospun in blends with PLLA. The g-CHWs/PLLA electrospun nanofibers exhibited advanced mechanical properties, and promoted the attachment, spread, and proliferation of MC3T3-E1 cells more effectively than PLLA nanofibers, suggesting their potential for utilization in bone regeneration applications [142].

The electrospinning of chitosan has attracted enormous scientific interest, and there are numerous reports concerning the development of chitosan-based electrospun nanofibers for various applications [143,144,145,146,147,148]. Pure electrospun chitosan nanofibers have been successfully generated from acidic solutions. In most cases, chitosan is electrospun using TFA as a solvent because it forms a salt with the amino groups of chitosan that reduces the intermolecular interactions between chitosan chains, facilitating its electrospinnability. The addition of CH_2_Cl_2_ in the solvent system has also been reported to improve the morphology of the nanofibers [143,147]. Sangsanoh et al. (2010) fabricated smooth nanofibers without beads from pure chitosan using TFA/CH_2_Cl_2_ as the spinning solvent system [149]. Nevertheless, electrospinning of pure chitosan is quite challenging due to the polyelectrolyte nature of the polysaccharide in acidic conditions. Under the application of an electric field, repulsive forces of charged species in the polymer backbone arise, which often lead to the formation of particles [150]. Chemical modifications of chitosan have been conducted to impart the desired function and properties and to facilitate its electrospinnability. After deacetylation of chitin, chitosan possesses amino and hydroxyl functional groups that can be modified to alter the properties of chitosan [21]. Grafting of synthetic polymers—such as PEG, poly(lactic-co-glycolic acid) (PLGA), and PCL—onto the chitosan backbone has been widely explored to improve the solubility of chitosan and develop hybrid materials of natural and synthetic polymers [151,152,153,154,155]. In this respect, PEGylated chitosan was successfully synthesized, exhibiting adequate solubility in water, THF, DMSO, DMF, and CHCl_3_. Electrospinning of PEGylated chitosan was accomplished in THF/DMF by the addition of Triton-X-100, affording uniform nanofibers [151]. Moreover, quaternary ammonium salts of chitosan have been designed via the reaction of the amino groups of chitosan with glycidyl trimethyl ammonium chloride. Quaternized chitosan is water-soluble, displaying superior antibacterial properties against *E. coli* and *S. aureus* in comparison to chitosan. Alipour et al. (2009) prepared quaternized chitosan/PVA electrospun nanofibers [156]. The carboxymethylation of the amino and hydroxyl groups of chitosan generates a water-soluble derivative of chitosan, and electrospun nanofibers of carboxymethyl chitosan have been developed successfully, with very promising results for biomedical applications due to their enhanced hydrophilicity, biocompatibility, and hemocompatibility [157,158].

Post-treatment of chitosan nanofibers is often required when chitosan is electrospun from solvent systems that contain TFA or acetic acid. The amino groups of chitosan form acetate salts that dissolve in aqueous solutions, with the resulting nanofibers losing their stability in neutral or weakly basic aqueous environments. Hence, a neutralization process is often required to remove the salts by submerging chitosan nanofibers in basic solutions, including saturated Na_2_CO_3_ and K_2_CO_3_ [135,159,160]. Indeed, it has been reported that neutralization of chitosan nanofibers with saturated Na_2_CO_3_ preserved their fibrous structure after continuous immersion in phosphate-buffered saline (PBS) or water for 12 weeks [159]. An alternative neutralization method has been reported recently by Fadaie et al. (2019), who fabricated chitosan/PEO nanofibers from acetic acid solutions and stabilized them upon exposure to water vapors for 30–120 min at 40–70 °C, enhancing their structural integrity in aqueous media [161]. As mentioned above, chitosan can form polyelectrolyte complexes with anionic polysaccharides (such as alginates) that are stable in aqueous media. In this context, Garcia et al. (2020) prepared electrospun nanofibers of chitosan/hyaluronan polyelectrolyte complexes with PEO. Heat treatment was applied to stabilize the fibers, and then PEO was removed by rinsing the fibers with EtOH. The obtained nanofibers presented reduced solubility at pH 7.4 and enhanced biocompatibility [162].

**Figure 7:**
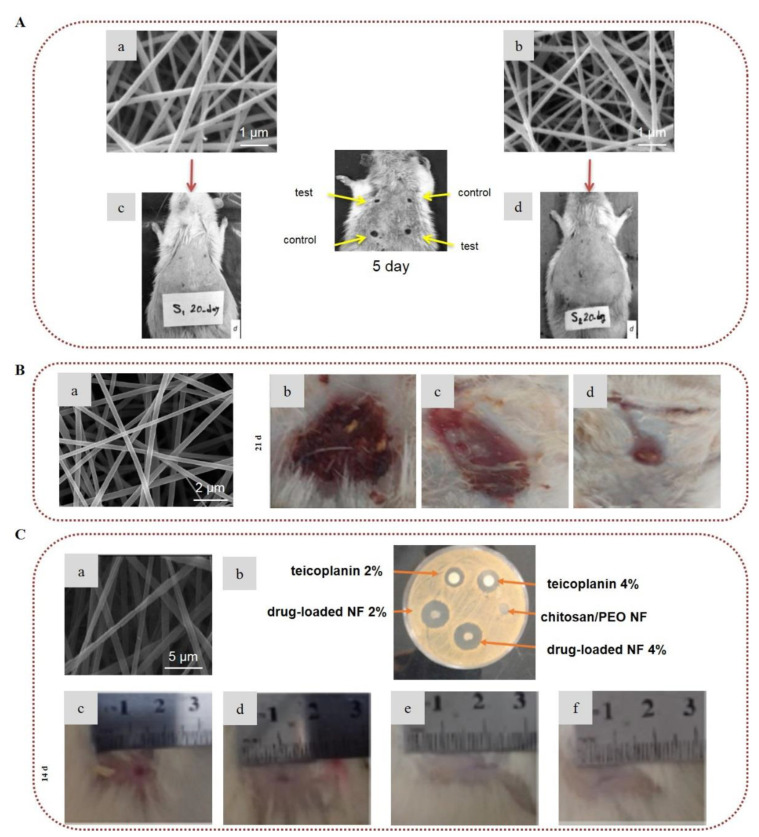
Chitosan-based electrospun nanofibers for wound healing applications: (**A**) SEM images of (**a**) chitosan/PVA nanofibers and (**b**) PCL/chitosan/PVA nanofibers. Images of the in vivo study in diabetic dorsum skin wounds treated with (**c**) chitosan/PVA and (**d**) PCL/chitosan/PVA (test (upper-left and lower-right wounds) and control (upper-right and lower-left wounds)). Reprinted/adapted with permission from [168], Copyright 2016, John Wiley and Sons. (**B**) (**a**) SEM image of 2% bromelain-loaded chitosan nanofibers. Images of the in vivo study of burn wounds in rat models treated with (**b**) control, (**c**) chitosan nanofibers, and (**d**) bromelain-loaded chitosan nanofibers on day 21. Reprinted/adapted with permission from [178], Copyright 2019, Elsevier. (**C**) (**a**) SEM image of 2% teicoplanin-loaded chitosan/PEO nanofibers. (**b**) Image of the antibacterial activity of teicoplanin, chitosan/PEO nanofibers, and teicoplanin-loaded chitosan/PEO nanofibers against *S. aureus*. Images of the in vivo wound healing study on rat models treated with (**c**) normal saline, (**d**) chitosan/PEO nanofibers, (**e**) teicoplanin-loaded 2%, and (**f**) teicoplanin-loaded 4% on day 14. Reprinted/adapted with permission from [186], Copyright 2020, Elsevier.

Chitosan has been vastly exploited for the treatment of acute and chronic wounds, because it can stimulate hemostasis and induce tissue regeneration [163,164]. Furthermore, it exhibits antimicrobial activity against a wide variety of bacteria and fungi due to its cationic nature, which is influenced by the degree of deacetylation and molecular weight [165,166]. Already, chitosan-based wound dressings exist commercially in the form of hydrogels, sponges, films, and nonwovens [164]. The wound healing potential of chitosan-based electrospun nanofibers has been examined in numerous reports [144,167]. Gholipour-Kanani et al. (2016) developed chitosan/PVA and PCL/chitosan/PVA nanofibrous scaffolds, and evaluated their healing efficacy on diabetic wounds. The in vivo study conducted on diabetic dorsum skin and diabetic foot wounds on male Sprague–Dawley rats demonstrated that both scaffolds provided faster wound closure as compared to the control, and afforded complete healing after 20 days (Figure 7A) [168]. In another study, chitosan/PEO electrospun nanofibers exhibited significant antibacterial activity against *S. aureus* due to the presence of chitosan [169].

Various bioactive compounds have been encapsulated into chitosan-based electrospun nanofibers to develop multifunctional scaffolds for enhanced antimicrobial protection and accelerated healing [170,171,172,173,174,175,176]. Curcumin–chitosan/PVA core–shell nanofibers with a polymer-free core were developed via coaxial electrospinning for wound healing and tissue engineering applications. Curcumin served as the core, while chitosan/PVA served as the shell layer. The release profile study showed that the core–shell nanofibers presented a controlled release of curcumin. In the antibacterial activity evaluation, the nanofibers were highly effective against *S. epidermidis* and MRSA *S. aureus—*which are resistant to β-lactam antibiotics—and also provided protection against *E. coli* and *P. aeruginosa* [177]. In another study, bromelain-loaded chitosan/PEO nanofibers tested for burn wound treatment showed great healing efficacy in vivo on rats (Figure 7B) [178]. PLA/chitosan nanofibers incorporating cod liver oil exhibited enhanced healing properties and faster wound closure in diabetic male rats as compared to pure PLA/chitosan nanofibers or pure cod liver oil [179].

Following another approach for the development of wound dressings, the controlled delivery of growth factors to the wound site has been also explored [180,181]. Piran et al. (2018) reported that nanofibers of PCL blended with chitosan nanoparticles incorporating the growth factors PDGF-BB, which are of high importance in the healing process, promoted the migration and proliferation of fibroblast cells to the injury site [180]. Ahmed et al. (2018), combining the healing properties of chitosan/PVA nanofibers with the antibacterial properties of ZnO, demonstrated that ZnO-loaded chitosan/PVA nanofibrous scaffolds constitute promising dressings for healing diabetic wounds. The ZnO-loaded nanofibers exhibited higher antibacterial activity against *E. coli, P. aeruginosa, Bacillus subtilis*, and *S. aureus*, as well as enhanced antioxidant activity, in comparison to neat chitosan/PVA nanofibers. Histological examination of wounds in diabetic rabbits revealed that the ZnO-loaded chitosan/PVA nanofibers provided accelerated healing of diabetic wounds [182]. Recently, the enzyme actinidin was successfully immobilized into chitosan/PEO electrospun nanofibers in order to evaluate their efficacy for the treatment of third-degree burns. The in vivo study on male Wistar rats confirmed the efficacy of the enzyme-immobilized nanofibers in the healing of burns, since they provided fast wound closure by preventing infection and bleeding. Histological examination demonstrated that the scaffolds promoted angiogenesis, epithelization, and collagen deposition, and prevented scar formation [183]. Chitosan-based nanofibrous wound dressings incorporating APIs have also been developed to deliver the active agents to the injured site and prevent infections [184,185,186]. Amiri et al. (2020) developed a local antibiotic delivery system made of chitosan and PEO nanofibers loaded with teicoplanin. The produced nanofibers were able to release teicoplanin for up to 12 days, almost doubling the antibacterial efficacy of the antibiotic. An in vivo study on male Wistar rats revealed that the nanofibers enhanced the rate of wound closure, suggesting a synergistic antibacterial effect with teicoplanin (Figure 7C) [186].

Over the last several years, chitosan-based electrospun nanofibers have been extensively investigated for the development of drug delivery systems [146,148,187]. Lancina et al. (2017) fabricated insulin-loaded chitosan/PEO electrospun nanofibers for the administration of insulin via the buccal mucosa. The encapsulated insulin retained its bioactivity after the electrospinning process, and its buccal permeability—as compared to that of free insulin—was remarkably improved when increasing the chitosan content [188]. Mendes et al. (2016) prepared electrospun nanofibers of chitosan blended with phospholipids, and studied the incorporation and release of curcumin, diclofenac, and vitamin B12 from the fibrous mats. Interaction between the amine groups of chitosan and the phospholipids resulted in increased stability of the nanofibers in PBS for at least one week, highlighting their potential application as transdermal drug delivery patches [189]. Aiming to achieve a sustained drug release profile, Jalvandi et al. (2017) conjugated levofloxacin with low molecular weight chitosan through covalent bonding of levofloxacin’s carboxyl groups with the amine groups of chitosan, and successfully electrospun the conjugate with PVA. The release rate study indicated that the burst release of the drug was significantly reduced as compared to that of the neat PVA/levofloxacin nanofibers, and a controlled release was attained [190]. Zupančič et al. (2019) developed bilayer nanofibrous mats of ciprofloxacin-loaded chitosan/PEO and metronidazole-loaded PCL for the local treatment of periodontal disease. To evaluate the efficacy of the nanofibers, a microflow apparatus that mimics the in vivo conditions of the periodontal pocket was utilized. The incorporated drugs presented a sustained release, exhibiting activity against *E. coli* and the periodontal pathogen *Aggregatibacter actinomycetemcomitans* [191]. Recently, a membrane composed of chitosan/silk protein electrospun nanofibers, incorporating 5-fluorouracil, was prepared for the inhibition of subconjunctival fibrosis in pterygium and antiglaucoma surgery. The fibrous membrane exhibited a sustained release of 5-fluorouracil in vitro and a prolonged inhibitory effect on subconjunctival myofibroblasts in vivo [192]. Magnetic Fe_3_O_4_ nanoparticles crosslinked or encapsulated into chitosan nanofibers were prepared for hyperthermia treatment of malignant tumors, since tumor cells are more sensitive to temperature than normal cells. Under the application of an alternating magnetic field, the composite fibers increased the cells’ incubation medium temperature to 45 °C and remarkably decreased the proliferation of the tumor cells in vitro [193].

Furthermore, innovative drug carrier systems based on chitosan nanofibers have been reported, demonstrating great potential for the controlled delivery of anticancer drugs to tumors, while minimizing the side effects on healthy tissues [148,194,195,196,197,198,199,200]. Zhu et al. (2019) developed core–shell electrospun nanofibers of poly(*N-*vinyl-2-pyrrolidone) (PVP) loaded with 5-fluorouracil as the core and PCL/chitosan as the shell for the transdermal treatment of skin melanoma. The in vitro study showed that the rapid release of 5-fluorouracil induced the apoptosis of B16F10 cancer cells and inhibited their proliferation. Furthermore, the controlled dissolution of chitosan from the scaffold protected normal skin (L929) cells from the side effects of 5-fluorouracil, suggesting that the fibers could provide a promising strategy for skin cancer therapy [196]. In another study, chitosan/gellan electrospun nanofibers incorporating resveratrol were successfully designed for targeted delivery to the gastrointestinal tract. The drug release study showed that half of the incorporated resveratrol was delivered to the intestine region. The encapsulation of resveratrol in the nanofibers enhanced its antioxidant activity as compared to that of free resveratrol, whereas the nanofibers displayed the same cytotoxicity levels with free resveratrol against HT29 cancer cells [197]. In a recent investigation, a pH-responsive drug delivery system of curcumin-loaded PLGA/chitosan nanofibers was developed for targeted cancer treatment. The encapsulation of curcumin provided enhanced antioxidant and antitumor activity in vitro against HT-29 cells. In addition, the release profile study showed that the release of curcumin increased at pH 2.0, with the fiber mats exhibiting the highest anticancer activity in acidic media [199].

In addition, chitosan electrospun nanofibers have been proposed as ideal candidates for the development of tissue engineering scaffolds [145,201,202,203,204]. Figueira et al. (2016) fabricated a novel layer-by-layer nanofibrous scaffold of PCL–hyaluronic acid/chitosan–zein loaded with salicylic acid for skin tissue regeneration. PCL–hyaluronic acid nanofibers were used as the outer layer for mechanical support, while the chitosan-based nanofibers were used as the inner layer to provide anti-inflammatory and antimicrobial properties. The bilayer membrane presented an initial burst release of salicylic acid sufficient to eliminate bacterial growth at the wound site, followed by a sustained release adequate to prevent infections at the wound. Furthermore, the fabricated scaffold induced the migration, adhesion, and proliferation of normal human dermal fibroblasts (NHDFs), accelerating the healing process, and indicating that the electrospun nanofibrous bilayer membrane could serve as a promising wound dressing system [205]. In another study, biomimetic nanofibrous scaffolds of chitosan/PCL coated with hydroxyapatite were designed for the regeneration of tendons and ligaments. The incorporation of hydroxyapatite enhanced the mechanical properties of the scaffolds, and induced higher attachment and proliferation of human osteoblast cells [206]. Recently, a novel tissue-engineered trachea of chitosan/PCL electrospun nanofibers layered on a 3D-printed biotracheal construct was successfully developed. The biotracheal construct consisted of a PCL frame and a bioink of collagen hydrogel incorporating chondrocytes. The nanofibrous membrane was assembled as a protective layer on the biotracheal graft to protect the collagen bioink, enhancing cartilage reconstruction. In vivo implantation in male Sprague–Dawley rats for two weeks demonstrated that the prepared biotracheal scaffold decreased the risk of tracheal collapse, and exhibited improved chondrogenic performance, indicating that such protective tissue-engineered tracheal grafts show great potential for cartilage regeneration [207].

**Figure 8:**
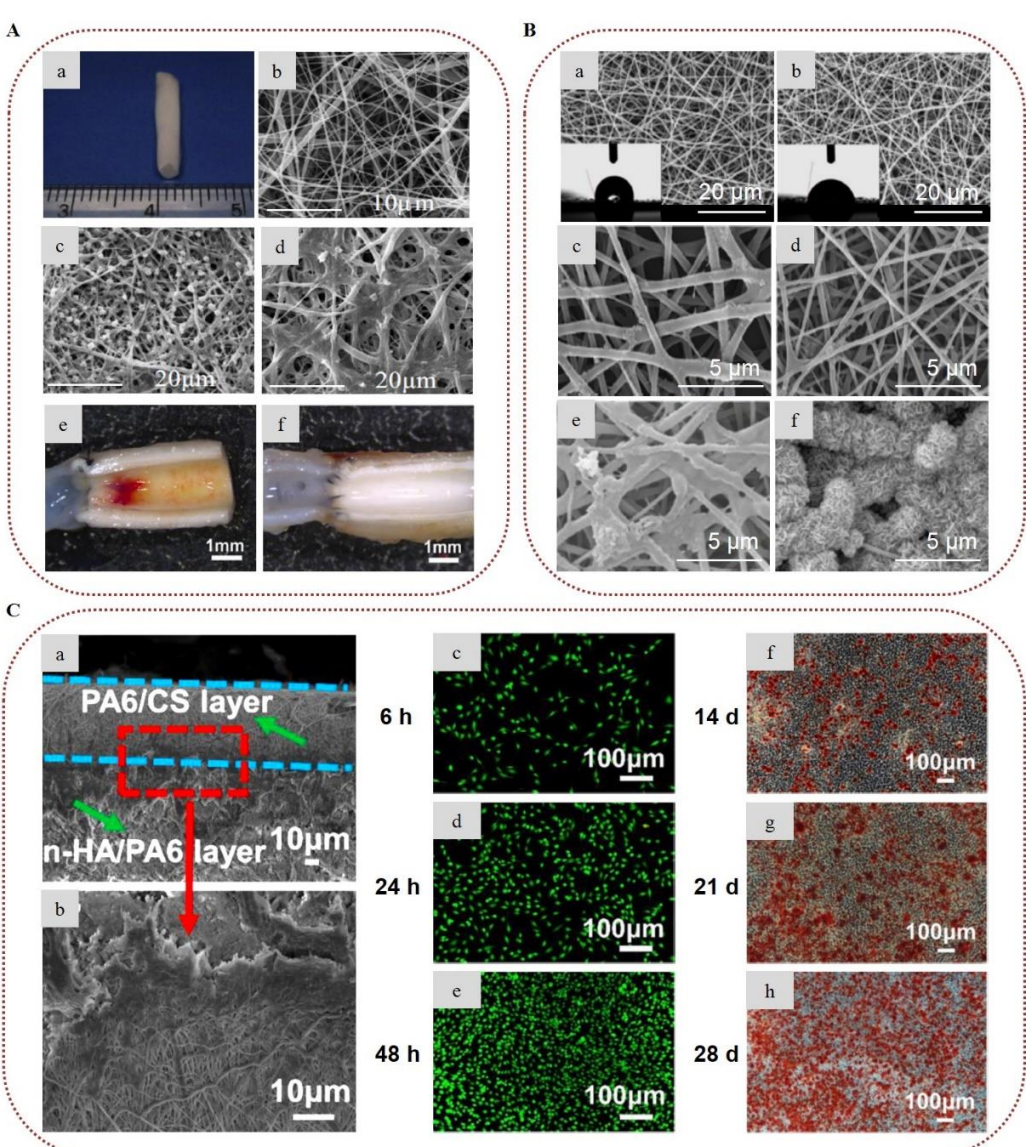
Chitosan-based electrospun nanofibers for tissue engineering applications: (**A**) (**a**) Image of a trilayer PCL/chitosan hybrid tubular graft. SEM images of (**b**) PCL/chitosan (5/4 *w*/*w*) electrospun nanofibers. SEM images of platelets’ adhesion on the surface of PCL/chitosan (5/4 *w*/*w*) nanofibers (**c**) before and (**d**) after heparin functionalization. Stereomicroscopic images of the explanted vascular grafts after implantation for 1 month of (**e**) PCL/chitosan nanofibers and (**f**) heparin-loaded PCL/chitosan nanofibers. Reprinted/adapted with permission from [208], Copyright 2014, Elsevier. (**B**) SEM images of (**a**) chitosan/PHBV nanofibers and (**b**) chitosan/PHBV/hydroxyapatite nanofibers. Degradation of (**c**) chitosan/PHBV nanofibers and (**d**) chitosan/PHBV/hydroxyapatite nanofibers after 2 months. SEM images of (**e**) chitosan/PHBV nanofibers and (**f**) chitosan/PHBV/hydroxyapatite nanofibers after treating them with simulated body fluids for 30 days. Reprinted/adapted with permission from [213], Copyright 2015, Elsevier. (**C**) (**a**,**b**) SEM images of the cross-section of bilayer scaffolds of chitosan/polyamide-6 (PA6/CS) electrospun nanofibers reinforced with polyamide-6/nano-hydroxyapatite (*n*-HA/PA6) cast film. (**c**–**e**) Images of live/dead staining of MC3T3-E1 cells co-cultured with extract fluids on (PA6/CS)/(n-HA/PA6) bilayer scaffolds for 6, 24, and 48 h. Live cells are stained green (calcein-AM stain), whereas dead cells are stained red (ethidium homodimer-1 stain). (**f**–**h**) Images of alizarin red staining after osteogenic induction of MC3T3-E1 cells for 14, 21, and 28 days, showing an increasing number of calcium nodules with the increase in culture time. Reprinted/adapted with permission from [221], Copyright 2021, Elsevier.

Yao et al. (2014) formulated small-diameter vascular grafts of trilayer chitosan/PCL electrospun nanofibers incorporating heparin for cardiovascular tissue regeneration. Heparin was immobilized in the scaffold via ionic bonding with chitosan, with the amount of immobilized heparin being proportional to the content of chitosan. An initial burst release of heparin was observed, followed by a sustained release for at least one month due to the strong ionic bonding of heparin with chitosan. The encapsulation of heparin enhanced the hemocompatibility of the vascular grafts, and resulted in reduced platelet adhesion and prolonged coagulation time. Moreover, it induced the growth of endothelial cells, and suppressed the proliferation of vascular smooth muscle cells. In vivo implantation in the abdominal aorta of male Sprague–Dawley rats for one month revealed excellent anti-thrombogenic properties, indicating that the utilization of heparin could serve as a promising technique for the development of vascular grafts (Figure 8A) [208]. Recently, bilayer nanofibrous vascular grafts of carboxymethyl chitosan/PCL and chitosan/PCL exhibited antithrombotic activity due to the incorporation of carboxymethyl chitosan and the antibacterial properties of chitosan, and may serve as promising candidates to minimize the risk of restenosis and postoperative infections [209]. Liu et al. (2017) demonstrated the potential utilization of chitosan-based nanofibers for cardiac tissue regeneration. Aligned nanofibers of chitosan/PLA induced the formation of cardiac tissue by promoting the attachment, proliferation, and viability of cardiomyocytes, expressing high levels of α-actinin and troponin I [210].

Lau et al. (2018) developed artificial nerve-guidance channels from electrospun uniaxially aligned chitosan nanofibers. Chitosan nanofibers were crosslinked with genipin to enhance their structural stability and control their degradation time. Compared to neat chitosan nanofibers, genipin-treated nanofibers displayed enhanced attachment and proliferation of Schwann cells, which are crucial for the survival and function of neurons. The in vitro peripheral nerve regeneration study on dorsal root ganglion explants indicated a remarkable increase in neurite growth along the uniaxially aligned nanofibrous network [211]. Considering the stabilizing and reducing properties of chitosan for the formation of gold nanoparticles, Saderi et al. (2018) developed conductive scaffolds of gold nanoparticles embedded in chitosan/PCL nanofibers to improve the electrical signal cues between neural cells and induce peripheral nerve regeneration. The scaffolds promoted enhanced attachment and a high proliferation rate of Schwann cells, indicating their potential application for nerve tissue engineering [212].

Biocomposite nanofibrous scaffolds for bone regeneration were developed by Zhang et al. (2015), who electrospun a mixture of chitosan, hydroxyapatite, and PHBV. Due to the hydrophobic nature of PHBV, the nanofibrous structure of the matrix remained intact even after 60 days in PBS. Moreover, the presence of hydroxyapatite induced biomineral deposition on the surface of the fibers. The in vitro study showed that the nanofibers enhanced the proliferation of osteoblasts and increased the activity of alkaline phosphatase, suggesting the fabricated biocomposites as promising scaffolds for bone tissue engineering applications (Figure 8B) [213]. The use of graphene oxide in chitosan nanofibers for enhanced osteogenic properties has also been explored. In this respect, graphene-oxide-loaded PCL/chitosan/collagen nanofibrous scaffolds were synthesized by Aidum et al. (2019), with the fabricated scaffolds displaying enhanced cell attachment, proliferation, and osteogenic activity associated with the graphene oxide ratio [214], while chitosan nanofibers reinforced with graphene oxide nanosheets reported by Chen et al. (2019) exhibited superior mechanical properties, making them optimal for biomedical applications [215].

Extensive research has been conducted to develop guided bone and tissue regeneration membranes based on chitosan nanofibers for periodontal regeneration [216,217,218,219,220,221,222]. Jin et al. (2018) prepared electrospun nanofibers of chitosan blended with silver-ion-loaded calcium phosphate, and crosslinked them with vanillin. The presence of silver ions provided strong antibacterial activity against *Porphyromonas gingivalis* and *Streptococcus* *mutans*, while the incorporation of calcium phosphate enhanced apatite mineralization. The chitosan-based membranes proved to be cytocompatible with BMSCs, showing great potential for guided bone regeneration [218]. Recently, bilayer scaffolds of chitosan/polyamide-6 electrospun nanofibers reinforced with polyamide-6/nano-hydroxyapatite cast film were designed for periodontal bone regeneration. The scaffolds demonstrated improved mechanical properties due to strong molecular interactions and chemical bonding between the components of the nanofibers and the cast film. Furthermore, the bilayer scaffolds exhibited sufficient biocompatibility and osteoconductivity, suggesting their potential for bone regeneration applications (Figure 8C) [221].

### 3.2. Marine Collagen

Collagen is a complex polypeptide that represents the most abundant protein in animal and human bodies. It is the main structural component of the ECM in tissues and organs, including skin, bones, tendons, and blood vessels, supporting cell attachment, proliferation, and differentiation [223,224]. To date, 29 types of collagen have been identified, consisting of three polypeptide chains intertwined in a triple-helical structure [225]. Each chain consists of approximately 1000 amino acid residues that form a repeated sequence of glycine-X-Y, where X and Y are usually proline and hydroxyproline, respectively. Cattle and pigs are the main sources for collagen extraction. However, porcine- and bovine-derived collagens pose the risk of transmitting diseases, while their application is often limited by religious restrictions related to the consumption of porcine and bovine products. Over the last years, marine organisms have attracted research interest as a safer alternative source of collagen free of such constraints [224]. Marine collagen is usually type I, and can be isolated from the bones, skin, and scales of fish or the connective tissues of jellyfish, sea urchins, starfish, and sea cucumbers, among others [2]. The discards of the fishing industry have attracted significant interest as a sustainable and low-cost source for the extraction of collagen [226].

Marine collagen has been broadly investigated for tissue engineering and other biomedical applications due to its low immunogenicity [2,227], while extensive research has been conducted to develop collagen-based electrospun nanofibers for various biomedical applications [228]. Fish collagen nanofibers were prepared by Zhou et al. (2013), who successfully electrospun fish collagen from an HFIP spinning solution. The fish collagen electrospun nanofibers were able to support the attachment and growth of HEK-293 cells [229]. Choi et al. (2015) developed electrospun scaffolds of fish collagen/PCL for 3D cell culture and tissue engineering applications. The cytocompatibility study demonstrated that the nanofibers were able to promote the adhesion, spread, and proliferation of thymic epithelial cells, also triggering the expression of the corresponding genes and proteins [230]. Zhou et al. (2017) developed a guided tissue/bone regeneration membrane made of fish collagen/bioactive glass/chitosan nanofibers. The in vitro study revealed that the fiber mats were able to improve the viability of human periodontal ligament cells (hPDLCs) and induce their adhesion and spread, stimulating their osteogenic differentiation. The incorporation of collagen and chitosan into the nanofibers improved the hydrophilicity of the membrane, promoting cell attachment, while the incorporation of chitosan and bioactive glass provided antibacterial activity against *S. mutans*. Moreover, the in vivo experiments for the treatment of furcation defects in dogs revealed that the scaffolds were able to induce bone regeneration [231]. In another work, composite electrospun nanofibers of fish bone collagen/PVA incorporating graphene oxide nanoparticles presented enhanced biocompatibility in vitro and accelerated wound healing in vivo, indicating their potential as wound dressing materials [232]. For guided bone regeneration, nanofibrous scaffolds of fish collagen/PLGA loaded with nano-hydroxyapatite were developed by Jin et al. (2019). The scaffolds displayed improved mechanical strength and an increased degradation rate compared to those of neat PLGA nanofibers. Furthermore, the nanofibers exhibited improved cytocompatibility and osteogenic differentiation in vitro [233].

**Figure 9:**
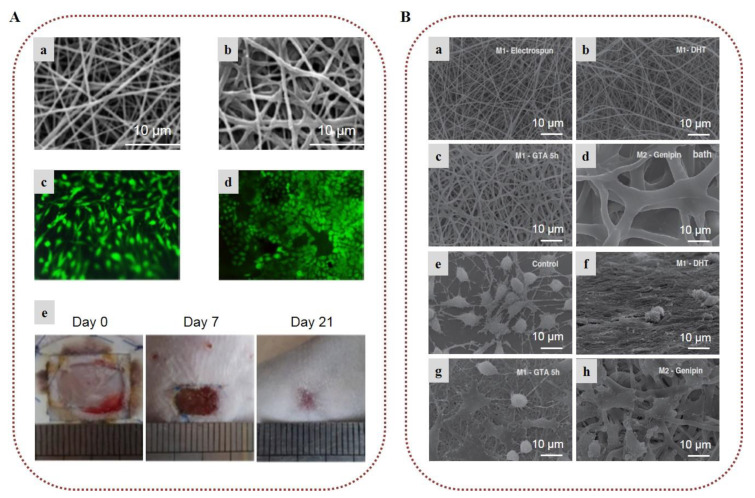
Fish collagen- and fish gelatin-based nanofibers for biomedical applications: (**A**) SEM images of fish collagen/PCL (9:1) electrospun nanofibers (**a**) before crosslinking and (**b**) after crosslinking with chitin oligosaccharides. Fluorescence micrographs of live/dead cell staining of (**c**) NHDF-neo and (**d**) HaCaT cells cultured on fish collagen/PCL (9:1) electrospun nanofibers crosslinked with chitin oligosaccharides after culturing for 7 days. Live cells are stained green (fluorescein diacetate stain), whereas dead cells are stained red (propidium iodide stain). (**e**) Images of the in vivo wound healing study on mice treated with the fish collagen/PCL (9:1) electrospun nanofibers crosslinked with chitin oligosaccharides on days 0, 7, and 21. Reprinted/adapted with permission from [235], Copyright 2021, Elsevier. (**B**) SEM images of fish gelatin electrospun nanofibers (**a**) before and after crosslinking (**b**) via dehydrothermal treatment, (**c**) with glutaraldehyde for 5 h, and (**d**) after genipin bath. SEM images of 3T3 fibroblasts’ cell viability on the surface of (**e**) control and crosslinked nanofibers with (**f**) dehydrothermal treatment, (**g**) glutaraldehyde, and (**h**) genipin bath. Reprinted/adapted with permission from [241], Copyright 2013, Elsevier.

Jia et al. (2020) demonstrated that electrospun nanofibers of fish collagen blended with PCL could be utilized for cartilage regeneration. The in vitro assessment confirmed the biocompatibility of the nanofibers by promoting chondrocyte proliferation and cartilage formation. Moreover, the in vivo study revealed that the fish collagen/PCL nanofibers induced cartilage regeneration. Increased concentrations of fish collagen worsened the mechanical properties, but resulted in better biodegradability and cell adhesion [234]. In a recent study, novel bilayer electrospun nanofibrous scaffolds composed of fish collagen and PCL at various ratios were fabricated and covalently crosslinked with chitin oligosaccharides. The produced fiber mats induced the adhesion, infiltration, and proliferation of fibroblast (NHDF-neo) and keratinocyte (HaCaT) cells in vitro, exhibiting rapid healing when implanted on a full-thickness wound in vivo, confirming their potential as skin implants for tissue regeneration applications (Figure 9A) [235].

### 3.3. Marine Gelatin

Gelatin is obtained through denaturation and hydrolytic degradation of collagen. The physicochemical characteristics of gelatin are influenced by the source (e.g., bovine, porcine, fish), collagen type, and manufacturing method. Depending on the treatment of collagen, two types of gelatin can be produced, namely, type A (acid treatment of collagen) and type B (alkali treatment of collagen). The resulting gelatin is a mixture of fractions varying in size that consists of glycine, proline, and hydroxyproline [236,237]. Over the last several years, extensive research has been conducted regarding the utilization of fish gelatin as a biomaterial devoid of religious restrictions and health-related concerns [238]. Usually, marine gelatin—especially from cold-water fish species—has poor rheological properties and forms less stable gels as compared to gelatin of terrestrial origin, due to its lower contents in proline and hydroxyproline [236]. Nonetheless, marine gelatin is a valuable biomaterial, since it exhibits antihypertensive, antioxidant, and antimicrobial activity, promotes wound healing and tissue regeneration, and has nutraceutical value as an important source of amino acids [238].

Over the last years, electrospun nanofibers based on marine gelatin have been developed for various applications. The first fabrication of marine gelatin electrospun nanofibers was reported by Songchotikunpan et al. (2008), who successfully prepared nanofibers from gelatin using either acetic acid or formic acid spinning solutions, investigating the effects of gelatin concentration on the fiber morphology. SEM analyses showed that at higher gelatin concentrations, smooth or mixed smooth and beaded fibers with increased average diameter were obtained [239]. Kwak et al. (2017) reported that aqueous solutions of cold-water fish-derived gelatin were successfully electrospun at room temperature, and uniform nanofibers were obtained. The fiber mats were crosslinked with glutaraldehyde to improve their structural integrity in water and their mechanical properties. The in vitro assessment indicated that the fish gelatin nanofibers displayed similar cytocompatibility to that of mammalian gelatin nanofibers [240]. Gomes et al. (2013) examined the influence of three different crosslinking techniques (glutaraldehyde, genipin, and dehydrothermal treatment) on the biocompatibility of fish gelatin nanofibers. Glutaraldehyde and genipin react with amino groups, and are widely used to stabilize nanofibers based on amino-group-containing polymers. Dehydrothermal treatment is a physical method during which water is removed from gelatin under high temperature and vacuum, resulting in intermolecular crosslinks. The viability study showed a faster proliferation rate of 3T3 fibroblast cells in the glutaraldehyde-crosslinked nanofibers as compared to genipin-crosslinked fibers, while the dehydrothermally treated scaffolds were the least biocompatible (Figure 9B) [241].

Composite electrospun nanofibers of fish gelatin reinforced with carboxylated nanodiamonds were developed for bone regeneration applications, triggering biomineralization and improving the adhesion and spread of MG63 osteoblast-like cells [242]. Gomes et al. (2017) developed nanofibrous scaffolds of fish gelatin blended with PCL and chitosan at various combinations. Gelatin/PCL nanofibers provided high cell adhesion, and the incorporation of chitosan improved the physical characteristics of the scaffold, demonstrating the potential of the blended fibers for tissue engineering applications [243]. Kwak et al. (2017) developed caffeine-loaded fish gelatin electrospun nanofibers with a high loading capacity, which were able to incorporate caffeine in its amorphous state. The fish gelatin nanofibers exhibited a rapid release of caffeine, showing great potential for their utilization in ultrafast delivery systems of hydrophilic bioactive ingredients [244]. Beishenaliev et al. (2019) developed fish gelatin nanofibers with improved stability in water via physical crosslinking upon UV irradiation. The irradiation did not affect the functional groups of gelatin, and the resulting nanofibers were biocompatible and induced the adhesion and proliferation of human keratinocytes. In the wound-scratch assay, the crosslinked fibers promoted rapid cell migration and enhanced wound closure, confirming their wound healing potential [245]. Wu et al. (2020) developed electrospun nanofibers of fish scale gelatin blended with modified PLA incorporating freshwater clam shell powder, with strong antibacterial efficacy against *E. coli* and *S. aureus*. The nanofibers exhibited improved cytocompatibility and mechanical properties [246].

### 3.4. Heparins and Heparan Sulfates

Heparins and heparan sulfates are sulfated polysaccharides that belong to the family of GAGs, and are present in the tissues of terrestrial and marine vertebrates and invertebrates. They are linear polysaccharides of complex structures and great heterogeneity, composed of alternating units of highly sulfated l-iduronic or d-glucuronic acid and d-glucosamine [247]. Heparins are composed mainly of l-iduronic acid, whereas heparan sulfates are predominantly composed of d-glucuronic acid. Furthermore, the d-glucosamine units of heparins are mainly *N*-sulfated, while in heparan sulfates they are mainly *N*-acetylated. Heparins are more sulfated, being produced mainly by mast cells, while heparan sulfates are produced by almost all cell types [248,249]. Heparin and heparan sulfate can interact with numerous proteins, regulating major biological processes such as cell proliferation, angiogenesis, and inflammation. Moreover, heparins are some of the most broadly used drugs due to their excellent anticoagulant activity [248,250]. Commercial heparins are usually extracted from porcine or bovine tissues, but due to the risks of zoonoses and religious restrictions, there is high demand for alternative sources [251]. Over the last several years, the isolation of heparins and heparan sulfates from marine organisms, such as mollusks, has been considerably investigated [251,252,253].

To the best of our knowledge, there are no reports yet on electrospun nanofibers from heparins or heparan sulfates extracted from marine organisms. However, their application in the form of electrospun nanofibrous systems might have great potential, especially considering that electrospun nanofibers based on mammalian heparin have attracted considerable interest as delivery systems and vascular tissue engineering scaffolds [20,119]. As an example, Wang et al. (2011) developed tubular nanofibrous scaffolds of gelatin/heparin as an inner layer and polyurethane as an external layer via bilayer electrospinning. The nanofibers were crosslinked with glutaraldehyde to retain their fibrous structure in aqueous media. The scaffolds possessed sufficient mechanical properties and displayed a prolonged release of heparin for over 14 days. The in vitro study showed that the nanofibers were hemocompatible, suppressed platelet adhesion due to the incorporation of heparin, and could be utilized as artificial blood vessels [254].

### 3.5. Chondroitin Sulfates

Chondroitin sulfates are linear sulfated polysaccharides that belong to the family of GAGs, and are present in the ECM of connective tissues, providing elasticity in the articular cartilage [131,255]. They are composed of repeating disaccharide units of glucuronic acid and *N*-acetyl-galactosamine linked through β-1,3 bonds [1]. The sulfate groups are usually positioned at C-4 or C-6 of the galactosamine residues [255,256]. Chondroitin sulfates can be isolated from a wide variety of terrestrial and marine vertebrates and invertebrates, including cattle, pigs, chicken, sharks, whales, sturgeon fish, and mollusks [1,131,257,258]. The chemical structures of chondroitin sulfates isolated from terrestrial and marine organisms differ in terms of molecular weight and sulfation. Chondroitin sulfates exhibit important biological activities, such as anticoagulant and antitumor properties. Furthermore, chondroitin sulfates are widely used for the treatment of osteoarthritis, owing to their anti-inflammatory activity and ability to hydrate tissues. Chondroitin sulfates have been broadly utilized in the design of chondrogenic and osteogenic scaffolds for wound healing and tissue engineering applications [131,255,256,257,259].

Nonetheless, there are limited reports concerning the electrospinning of chondroitin sulfates isolated from marine organisms. Jiang et al. (2019) developed composite nanofibrous scaffolds of either chondroitin sulfate isolated from shark cartilage or rat tail collagen blended with poly(PCL-polytetrahydrofuran urethane) for cartilage regeneration. Both scaffolds promoted the chondrogenesis of bone marrow-derived mesenchymal stem cells and increased the regeneration rate of the injured cartilage, highlighting the chondrogenic potential of marine-derived chondroitin sulfate [260]. In a recent study, uniform nanofibers of chondroitin sulfate isolated from shark cartilage were electrospun with collagen and crosslinked with 1-ethyl-3-(3-dimethyl-aminopropyl)-1-carbodiimide hydrochloride/*N*-hydroxy succinimide. The crosslinked fiber mats presented enhanced mechanical properties and stability against collagenase degradation. The in vitro study indicated that at low crosslinker concentrations the fiber mats presented adequate cytocompatibility, supporting the proliferation of epithelial cells [261].

### 3.6. Hyaluronans

Hyaluronans, also known as hyaluronic acids, are a group of non-sulfated GAGs present in the ECM of connective tissues in marine and terrestrial animals [259,262]. They are linear polysaccharides composed of alternating disaccharide units of *N*-acetyl-d-glucosamine and d-glucuronic acid linked through β-1,3 and β-1,4 glycosidic bonds, with molecular weights that range between 100 and 100,000 kDa [259,263]. Over the last years, the isolation of hyaluronans from marine sources—such as bivalves, stingray livers, fish cartilage matrix, and eyeball vitreous humor—has been well established [2,259]. Hyaluronans are viscoelastic and highly hygroscopic, and are involved in wound healing, enhancing tissue hydration, and resistance to mechanical damage. They are widely used in the biomedical field, finding applications in ophthalmology, orthopedics, aesthetic dermatology, and wound treatment [264,265,266,267].

Electrospinning of hyaluronans is very challenging due to their high solution viscosity and high surface tension [23,268]. Although, to the best of our knowledge, there are no reports yet on marine-derived hyaluronic acid nanofibers, their utilization in electrospun fibrous scaffolds is expected to be feasible and very promising, since in recent years there has been an increasing interest in hyaluronic acid-based electrospun nanofibers [20,23,119,268]. Recently, Abdel-Mohsen et al. (2019) developed electrospun nanofibers of hyaluronan/PVA loaded with silver nanoparticles. The incorporation of hyaluronan/silver nanoparticles significantly improved the mechanical strength of the nanofibrous mats as compared to that of pure PVA fibers [269]. Grimaudo et al. (2020) designed innovative ocular drug delivery systems of hyaluronan/PVP electrospun nanofibers encapsulating the antioxidant ferulic acid and crosslinked with the antimicrobial peptide ε-polylysine. Hyaluronan was used for the modulation of the release of ferulic acid to the ocular surface. The nanofibers exhibited a fast release of ε-polylysine and ferulic acid, possessing significant antibacterial activity against *P. aeruginosa* and *S. aureus*—probably due to the presence of ε-polylysine. Furthermore, the ocular compatibility study showed that the fiber mats were non-irritant, while being cytocompatible with fibroblasts [270].

## 4. Other Marine Biopolymers

Mauran is an exopolysaccharide produced by the halophilic bacterium *Halomonas maura* [271]. It is a sulfated polysaccharide with high uronic acid content, and is mainly composed of mannose, galactose, glucose, and glucuronic acid [272]. Aqueous solutions of mauran are highly viscous under a wide pH range, and show viscoelastic, thixotropic, and pseudoplastic behavior [271].

To date, only Raveendran et al. (2013) have reported on the electrospinnability of mauran. Electrospun nanofibers of mauran blended with PVA were successfully prepared, with a uniform structure and small average diameter. The biocompatibility of the nanofibers was confirmed in vitro on murine fibroblast cells and mesenchymal stem cells. In cell adhesion studies, the scaffolds were found to promote cell adhesion, migration, proliferation, and differentiation, showing significant potential for tissue engineering and other biomedical applications [273].

## 5. Future Trends and Conclusions

Electrospinning offers a versatile technological platform for the production of tailor-made advanced nanostructured systems for various applications in the biomedical field. The broad spectrum of bioactivities exhibited by marine biopolymers renders them ideal candidates for the development of novel nonwoven biomaterials with beneficial functionalities and desired micromechanical properties. Based on the studies highlighted in the present review, it is obvious that electrospun micro/nanofibrous matrices from marine biopolymers have great potential for utilization as biomaterials (e.g., wound dressings, scaffolds for cell proliferation and differentiation, adhesives, drug-release modifiers) for various biomedical applications (e.g., tissue engineering, drug delivery and controlled release). In addition to alginates and chitosan, which are extensively studied as marine biopolymers for the fabrication of electrospun biomedical scaffolds, the interest in the electrospinning of other marine biopolymers, such as carrageenans, ulvans, fucoidans, and marine collagen and gelatin, is steadily increasing. Future advancements regarding the electrospinning of marine biopolymers may include the utilization of yet-unexploited marine biopolymers for the production of nonwoven matrices, or in-depth investigation concerning the development of multifunctional fibrous biocomposites. The absence of polymer chain entanglements, the high surface tension, the high gelation, and the poor mechanical properties of marine biopolymers that constrict their utilization into electrospun fibers can be overpassed through chemical modifications, polyelectrolyte complexations, addition of surfactants, crosslinking treatments, coaxial electrospinning, and/or other advanced electrospinning techniques. The use of polymer blends, dispersions, or emulsions of marine biomaterials with synthetic polymers can enhance their electrospinning potential for the development of novel hybrid nanofibrous scaffolds with fascinating properties. The electrospun marine-based nonwovens resulting from the combination of different marine biopolymers with synthetic or other natural polymers can offer the advantageous properties of the chosen materials. Moreover, the incorporation of bioactive ingredients into electrospun marine-based fibers can lead to state-of-the-art multifunctional nanostructured biomaterials for drug delivery, wound healing, and tissue engineering applications.

## Data Availability

Not applicable.
